# Using simple artificial intelligence methods for predicting amyloidogenesis in antibodies

**DOI:** 10.1186/1471-2105-11-79

**Published:** 2010-02-08

**Authors:** Maria Pamela C David, Gisela P Concepcion, Eduardo A Padlan

**Affiliations:** 1 Virtual Laboratory of Biomolecular Structures, Marine Science Institute, College of Science, University of the Philippines Diliman, Quezon City 1101, Philippines

## Abstract

**Background:**

All polypeptide backbones have the potential to form amyloid fibrils, which are associated with a number of degenerative disorders. However, the likelihood that amyloidosis would actually occur under physiological conditions depends largely on the amino acid composition of a protein. We explore using a naive Bayesian classifier and a weighted decision tree for predicting the amyloidogenicity of immunoglobulin sequences.

**Results:**

The average accuracy based on leave-one-out (LOO) cross validation of a Bayesian classifier generated from 143 amyloidogenic sequences is 60.84%. This is consistent with the average accuracy of 61.15% for a holdout test set comprised of 103 AM and 28 non-amyloidogenic sequences. The LOO cross validation accuracy increases to 81.08% when the training set is augmented by the holdout test set. In comparison, the average classification accuracy for the holdout test set obtained using a decision tree is 78.64%. Non-amyloidogenic sequences are predicted with average LOO cross validation accuracies between 74.05% and 77.24% using the Bayesian classifier, depending on the training set size. The accuracy for the holdout test set was 89%. For the decision tree, the non-amyloidogenic prediction accuracy is 75.00%.

**Conclusions:**

This exploratory study indicates that both classification methods may be promising in providing straightforward predictions on the amyloidogenicity of a sequence. Nevertheless, the number of available sequences that satisfy the premises of this study are limited, and are consequently smaller than the ideal training set size. Increasing the size of the training set clearly increases the accuracy, and the expansion of the training set to include not only more derivatives, but more alignments, would make the method more sound. The accuracy of the classifiers may also be improved when additional factors, such as structural and physico-chemical data, are considered. The development of this type of classifier has significant applications in evaluating engineered antibodies, and may be adapted for evaluating engineered proteins in general.

## Background

Antibodies are used in a number of therapeutic procedures such as target-specific anti-cancer therapy, immunosuppression, and purging prior to bone marrow transplants. Most of those antibodies are of nonhuman origin, and their administration often results in the generation of adverse immune responses, which also limit their efficacy [1]. Humanization is usually performed to lessen the occurrence of these responses, to improve circulation half-life, and to restore effector functions [1,2]. Current humanization strategies include the retention of variable domains or the specificity-determining residues (SDR) only, grafting of complementarity-determining regions (CDR), and veneering [3-6].

Humanization, however, may decrease the thermal stability of an antibody and result in affinity reduction, as well as amyloid fibril formation, especially when the substitutions leave the humanized antibody prone to unfolding [3,7,8]. Studies indicate that the potential to form fibrils is a general property of polypeptide chains, but the propensity for amyloidosis is largely influenced by its sequence and the stability of its native state [9-11]. Furthermore, there is evidence that some antibody sequences, notably kappa light chain sequences, become prone to fibril formation due to point mutations acquired during affinity maturation [12]. Apart from these, events that lead to misfolding, such as conformational transitions between alpha helices and beta sheets, and partial or complete unfolding, could lead to amyloidosis [13-15]. Consequently, it would be of interest to develop a method to predict such events, as well as to identify mutations that could lead to amyloidosis. Currently, a number of computational methods are available for amyloidogenic potential prediction [16-18]. These generally use either the physicochemical properties of amino acids to create models for predicting aggregation rate on mutation and identifying hotspots, or the information from overlapping amyloidogenic polypeptide decomposition [17]. Recently, a method using mean packing density profiling has also been reported, and has been found to be able to predict both amyloidogenic and intrinsically disordered regions in both peptides and proteins [19]. Nevertheless, these methods yield predictions on which *regions *of a sequence are potentially amyloidogenic; for highly similar sequences, as the case is with both amyloidogenic and non-amyloidogenic antibodies, results from such methods are not so easy to distinguish (See Supplementary Information, additional file 1). In this paper, we explore the use of naive Bayesian and decision tree classification methods for predicting the amyloidogenic propensities of antibody sequences, with the primary application of predicting amyloidogenic propensities of engineered antibodies in mind. The naive Bayesian method provides the advantage of taking the effects of mutations at specific combinations of positions into account. The decision tree, on the other hand, intuitively allows the evaluation of more factors that may contribute to the amyloidogenic potential. For generating the classifiers in both methods, 143 amyloidogenic antibody sequences derived from twelve different germlines and 158 corresponding non-amyloidogenic derivatives were used. The unambiguous assignment of amyloidogenic and non-amyloidogenic sequences to their respective germlines is a critical premise in this paper. Germlines are DNA elements that define the basic, inherited antibody repertoire of an individual, which are rearranged and mutated during the response to foreign antigens [20]. As indicated previously, some sequences become prone to fibril formation after this mutation process [12]; consequently, the generation of separate alignments for the amyloidogenic and non-amyloidogenic derivatives of a single germline might lead to the identification of mutation patterns or characteristics exclusively associated with amyloidosis. It is critical that sequences are assigned correctly to a germline in order to ensure that the mutations observed are actual mutations, and do not arise from incorrect alignments. All alignments used in this paper are hand-annotated.

To test the classifiers and to evaluate the effects of the training set size, a holdout test set consisting of an additional 103 amyloidogenic sequences and 28 non-amyloidogenic sequences for eight of the twelve germlines was used. The naive Bayesian method, which is solely based on positional information, yields a prediction accuracy of 60.84% for amyloid-formers after LOO cross-validation, which is consistent with the 61.16% accuracy for the holdout test set. When the latter is included in the training set, LOO cross-validation accuracy increases to 81.08%. Sequences classified using a decision tree, on the other hand, yielded an average prediction accuracy of 78.64% for the holdout test set.

## Results

### A direct implementation of the Naive Bayesian method results in prediction accuracies between 60.84% and 81.08%

LOO cross-validation was performed to evaluate the accuracy of the Bayesian classifier; this particular method was used to allow the calibration data to be reused as test samples while simulating the prediction of future unknowns [21]. The average accuracy from this validation was at 60.84 ± 35.96% for classifying amyloidogenic sequences, with 25.95% of the non-amyloidogenic sequences being misclassified (Table 1, AMC and NAMC). Validation performed on the holdout test set yielded an average accuracy of 61.16 ± 13.75%, which falls within the LOO cross validation result (Table 1, AM Test).

**Table 1:** Naive Bayes classifier accuracy

Germline	AMC^1^		NAMC		AMC, new^2^		NAMC, new		AM Test^3^		NAM Test	
	*C*	*A*	*C*	*A*	*C*	*A*	*C*	*A*	*C*	*A*	*C*	*A*

**J00248**	5	8	13	15	N.A.	N.A.	N.A.	N.A.	N.A.	N.A.	N.A.	N.A.

**M30446**	0	6	7	10	N.A.	N.A.	N.A.	N.A.	N.A.	N.A.	N.A.	N.A.

**X72813**	0	6	18	19	1	8	19	19	1	2	N.A.	N.A.

**X93620**	12	22	12	16	31	33	15	16	9	11	N.A.	N.A.

**X93627**	6	12	14	14	17	19	13	14	4	7	N.A.	N.A.

**X93632**	0	5	8	9	N.A.	N.A.	N.A.	N.A.	N.A.	N.A.	N.A.	N.A.

**X93640**	6	11	10	13	9	17	12	13	4	6	N.A.	N.A.

**Z22188**	11	15	10	12	29	34	9	12	13	19	N.A.	N.A.

**Z22191**	0	5	9	9	N.A.	N.A.	N.A.	N.A.	N.A.	N.A.	N.A.	N.A.

**Z22197**	7	8	0	6	14	26	10	17	12	18	11	11

**Z22208**	7	13	12	14	31	35	13	20	8	22	4	4

**Z73673**	26	32	4	21	49	50	25	34	12	18	10	13

**Accuracy (%)**	**60.84 ± 35.96**		**74.05 ± 31.49**		**81.08 ± 29.32**		**77.24 ± 13.04**		**61.16 ± 13.75**		**89.28 13.32**	

^1 ^Classifiers generated with the original training set comprised of 143 amyloidogenic and 158 non-amyloidogenic sequences

^2 ^Classifiers generated with the original training set and the holdout test set

^3 ^Results using AMC/NAMC, i.e. old classifiers

C = Correct; A = Actual

To evaluate the effects of training set size, the holdout test set was combined with the original training set to generate a new set of classifiers. These were again subjected to LOO cross-validation, yielding a higher average accuracy of 81.08 ± 29.33% (Table 1, AMC, new).

### Germline-specific decision trees result in an average prediction accuracy of 78%

**Figure 1:**
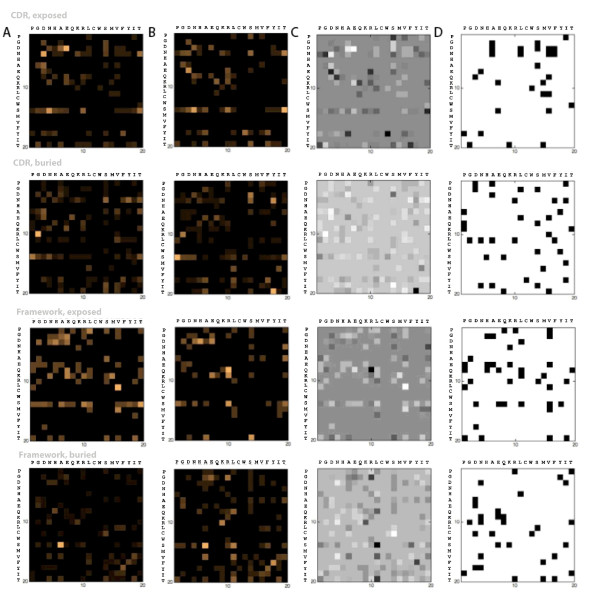
**Normalized mutation matrices of amyloidogenic (Column A) and non-amyloidogenic derivatives (Column B) of 12 antibody germlines**. Original residues are in rows and corresponding replacement residues are in columns. The amino acids have been arranged according to increasing *β*-sheet forming propensities [54]. The intensity matrix of the difference between the amyloidogenic and non-amyloidogenic matrices (Column C) reflects the relative predominance of a mutation type in either amyloid or non-amyloid formers. A fourth matrix set (Column D) is used to indicate the mutations that occur exclusively in amyloidogenic derivatives. Separate matrices were generated for mutations in buried CDR, exposed CDR, buried FR and exposed FR positions.

**Figure 2:**
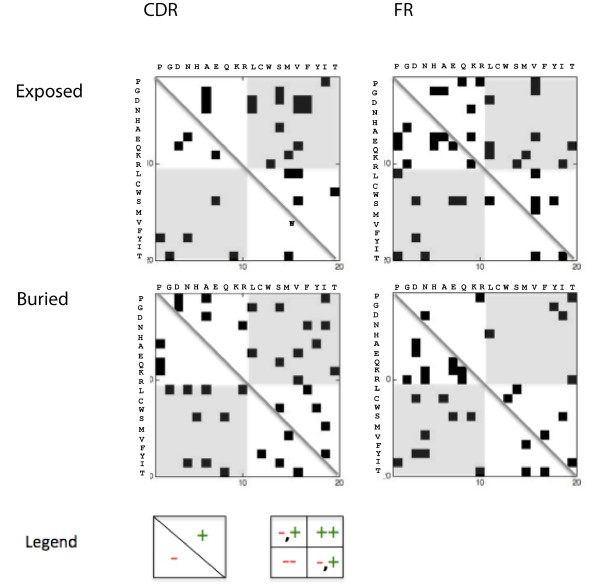
**Analysis of mutations exclusive to amyloidogenic derivatives**. A rough analysis of mutation patterns could be made by dividing the matrix using the diagonal, or by dividing it into quadrants. Mutations to the right of the diagonal are characterized by increased sheet-forming propensities (+), while those to the left imply the opposite (-). In terms of the quadrants, which are numbered in the same way as the Cartesian plane, the first contains information on mutations from low- to mid-propensity, sheet-associated amino acids to relatively high-propensity sheet-associated amino acids (++), while the third quadrant contains the opposite (--). In the most general sense, mutations either on the right of the diagonal, or in the first and third quadrants (shaded), would be the biggest contributors to destabilization. The analysis indicates that a significant number of mutations in the exposed CDR residues result in increased *β*-sheet-forming propensities, while mutations in buried FR residues tend to be associated with a decrease in *β*-sheet-forming propensities.

**Figure 3:**
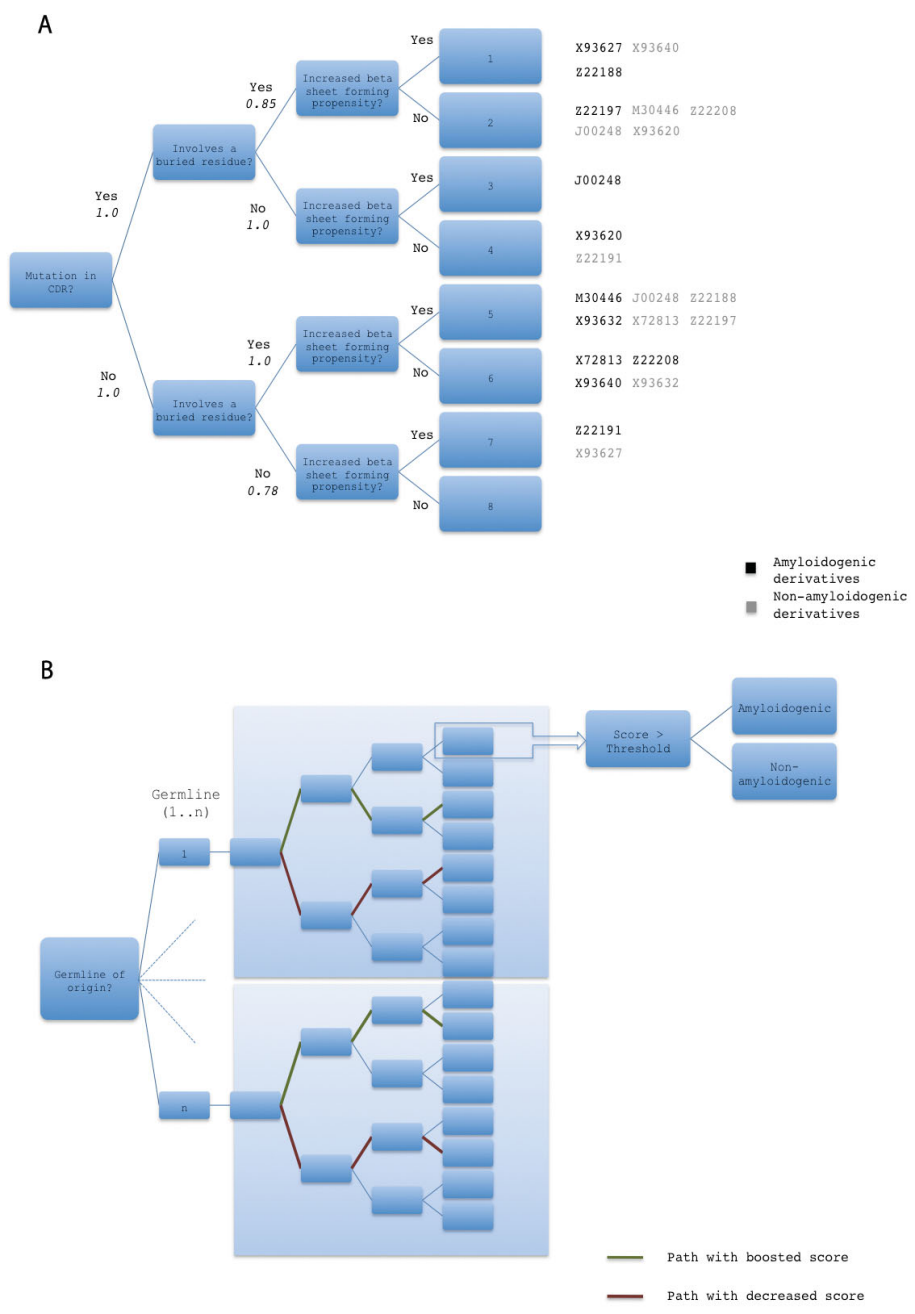
**Decision tree for the evaluation of individual mutations**. A decision tree (A) was constructed in order to evaluate the contribution of a mutation to amyloidogenicity. A *path *is followed for each mutation, depending on its position and exposure, as well as on the increase or decrease in sheet-forming propensity associated with it. Each path leads to one of eight terminal nodes, which is associated with a *score*, defined as the product of the weights (in italics) along the path leading to it. An analysis of paths taken by amyloidogenic and non-amyloidogenic derivatives of the different germlines indicated that different pairs of terminal nodes may be used to provide maximum separation between these derivatives. For instance, amyloidogenic derivatives of X93627 mostly end in leaf 1, while the non-amyloidogenic counterparts are more frequently associated with leaf 7; germline derivatives that can be distinguished using specific terminal nodes are indicated in the illustration. Based on this analysis, a final tree (B) was created which branches first on the basis of the germline to which the derivative being tested belongs; the structure and weights of the original tree (A) are kept. Each edge emanating from a germline node is connected to a copy of the original tree, where weights on paths which could be used for maximizing the separation between amyloidogenic and non-amyloidogenic derivatives are either boosted or decreased tenfold. For the illustrative example in (B), paths for J00248 (Germline 1) and Z22208 (Germline n) are shown.

In order to construct a decision tree, we analyzed the nature of the mutations exclusively associated with amyloid formers using an algorithm and accompanying visualization program that we have previously developed [22,23]. Results indicate that most of the mutations that occur exclusively in CDR residues or in FR residues of amyloidogenic derivatives are most likely the biggest contributors to misfolding, with 69% of the mutations in exposed CDR resulting in a general increase in sheet-forming propensity, as opposed to the 36% in buried FRs (Figures 1 and 2; Table 2). In contrast, the complements (31% for exposed CDRs and 64% for buried FRs) resulted in decreased sheet-forming propensities. We used these information as branch weights for an initial decision tree (Table 3); before establishing the weight thresholds for classification, however, we checked if paths taken by amyloidogenic and non-amyloidogenic derivatives can be generalized. Interestingly, we found no consensus paths for either amyloidogenic or non-amyloidogenic sequences; instead, consensus paths appear to exist for each germline (Figure 3A, Table 4). Consequently, we constructed a second decision tree which takes the germline of origin into account, as the case was in the Bayesian analysis. Depending on the germline, weights along selected paths are either boosted or decreased (Figure 3B, Table 4). Thresholds for separation were chosen to maximally distinguish samples in the training set (Table 5), and are evaluated using the holdout test set. Table 6 lists the classification results per germline.

**Table 2:** Summary of mutations exclusive to amyloid formers

Exposure, Region	Increased *β*-sheet- forming propensity	Decreased *β*-sheet- forming propensity
Exposed CDR	20	9

Exposed FR	20	19

Buried CDR	21	16

Buried FR	12	21

**Table 3:** Decision tree weights

Edge	Weight	Reference for weight
CDR	1.0	Ratio of CDR:FR mutations

FR	0.79	

CDR - exposed	0.78	Ratio of buried:exposed CDR mutations

CDR - buried	1.0	

FR - exposed	1.0	Ratio of buried:exposed FR mutations

FR - buried	0.85	

CDR - exposed - Δ	0.69	Ratio of mutations increasing (Δ) sheet-forming propensities to mutations decreasing (▽) sheet-forming propensities in exposed CDR residues

CDR - exposed - ▽	0.31	

CDR - buried - Δ	1.00	Ratio of mutations increasing (Δ) sheet-forming propensities to mutations decreasing (▽) sheet-forming propensities in buried CDR residues

CDR - buried - ▽	0.76	

FR - exposed - Δ	1.00	Ratio of mutations increasing (Δ) sheet-forming propensities to mutations decreasing (▽) sheet-forming propensities in exposed FR residues

FR - exposed - ▽	0.95	

FR - buried - Δ	0.74	Ratio of mutations increasing (Δ) sheet-forming propensities to mutations decreasing (▽) sheet-forming propensities in buried FR residues

FR - buried --▽	0.43	

**Table 4:** Summary of leaves providing maximum separation between amyloidogenic and non-amyloidogenic derivatives of different germline sets*

Leaf	J00248	M30446	X72813	X93620	X93627	X93632	X93640	Z22188	Z22191	Z22197	Z22208	Z73673
1	0.091	0.009	0.024	-0.016	0.042	0.046	-0.001	0.044	0.028	0.036	-0.032	-0.036

2	-0.030	0.008	0.009	-0.013	*-0.135*	-0.093	0.022	-0.075	**0.073**	0.089	0.052	**0.062**

3	-0.038	-0.001	**0.071**	-0.035	-0.038	*-0.209*	**0.100**	-0.085	-0.035	-0.017	**0.068**	0.003

4	*-0.058*	**0.030**	*-0.145*	-0.017	0.053	**0.116**	-0.008	*-0.123*	0.058	*-0.198*	**0.039**	0.014

5	-0.044	0.007	0.056	**0.065**	0.018	0.070	-0.009	-0.081	*-0.092*	-0.057	-0.025	0.008

6	**0.132**	-0.028	0.043	0.004	0.026	0.070	0.012	0.079	0.058	0.026	-0.006	-0.029

7	*-0.058*	*-0.031*	-0.054	*-0.052*	-0.048	0.000	-0.018	0.102	-0.082	**0.158**	*-0.105*	*-0.016*

8	0.007	0.006	0.066	0.063	**0.083**	0.00	*-0.099*	**0.139**	-0.011	-0.037	0.009	0.040

* Values were obtained by substracting the percentage of mutations in non-amyloidogenic derivatives from the percentage of mutations in amyloidogenic derivatives terminating in a given leaf. Minimum and maximum values per germline set, which were used to identify the paths where scores were decreased and boosted, respectively, are shown in italics and boldface, respectively.

**Table 5:** Summary of thresholds

Germline	Threshold
J00248	1.70

M30446	1.50

X72813	1.75

X93620	0.65

X93627	0.85

X93632	1.80

X93640	2.50

Z22188	0.80

Z22191	0.75

Z22197	0.65

Z22208	1.50

Z73673	0.75

**Table 6:** Decision tree classification accuracy*

Germline	AM		NAM	
**J00248**	N.A.	N.A.	N.A.	N.A.

**M30446**	N.A.	N.A.	N.A.	N.A.

**X72813**	1	2	N.A.	N.A.

**X93620**	9	11	N.A.	N.A.

**X93627**	7	7	N.A.	N.A.

**X93632**	N.A.	N.A.	N.A.	N.A.

**X93640**	3	6	N.A.	N.A.

**Z22188**	14	19	N.A.	N.A.

**Z22191**	N.A.	N.A.	N.A.	N.A.

**Z22197**	13	18	9	11

**Z22208**	19	22	3	4

**Z73673**	15	18	9	13

**Average accuracy **(%)	78.64 ± 17.44		78.64 ± 6.30	

* N.A. indicates that no additional sequences were obtained for this germline.

## Discussion

The diversity of the antibody repertoire is generated through the combinatorial recombination of a small pool of germline genes and its somatic hypermutation. Nevertheless, these diversification processes have setbacks, including the generation of autoreactive antibodies as well as structurally compromised antibodies [24]. The latter are implicated in diseases that range from benign, high-level soluble light-chain production to pathological deposition in glomerular basal membrane cells, bone marrow plasma cells, interstitial tissues, arterial walls and basement membranes [24,25]. These unwanted effects often result from a set of mutations whose consequences on the structure are not so evident, so much so that the resulting unstable light chains evade elimination during posttranslational quality control [24,26]. Avoiding such mutations or combinations thereof is critical in antibody engineering.

From studies carried out on amyloidogenic antibodies, some patterns that can be linked to amyloidosis have been found. Poshusta and co-workers, for instance, have reported that non-conservative mutations account for 0.6 - 0.79 of the total mutations in V_*λ *_sequences, while 0.4 - 0.59 account for the mutations in V_*κ *_sequences [27]. They also reported differences in the location of these mutations in patients with different secreted levels of light chains. Specifically, it is implied that the position of mutations, and not the amount secreted, plays a more important role in light chain amyloidogenic propensity, based on studies on patients with very low light chain levels but advanced amyloid deposition [27]. Consequently, it is clear that two factors, at the minimum, have to be considered in generating a protocol for predicting amyloid formation: the combination of positions at which the mutation occurs, as well as how these affect the structural stability of the antibody.

A review by Caflisch [17] classified the computational approaches used in predicting protein and peptide aggregation propensity into two general groups. The first makes use of the physicochemical properties of the amino acids to create phenomonological models for predicting aggregation behavior on mutation. The second, on the other hand, uses the decomposition of amyloidogenic peptides into overlapping segments. These are then simulated to the level of atoms to obtain estimates of aggregation propensity, as well as the structural details of the aggregates. Some programs that have since been developed to deal with amyloidosis include the PASTA server [28,29], a fibril prediction program [30], AGGRESCAN [16], Zyggregator [31], and Pafig [32], among others. Nevertheless, these algorithms deal with the prediction of the segments involved or possibly involved in amyloidosis, but do not generate direct predictions on whether a given sequence will be amyloidogenic or not. Here, we propose methods that may be used to complement existing prediction protocols in obtaining direct predictions about the amyloidogenicity of an antibody sequence; the method may be extended to other protein types, provided that there are sufficiently related positive and negative training sets.

A Naive Bayesian classifier uses probabilities to link hypotheses to events defined by a set of attributes. In Mitchell [33], the Naive Bayesian classifier *v*_*N*_*B *is defined as:(1)

where *v*_*j *_is one of a set of V classes and *a_i _*is one of *n *attributes describing an event.

**Figure 4:**
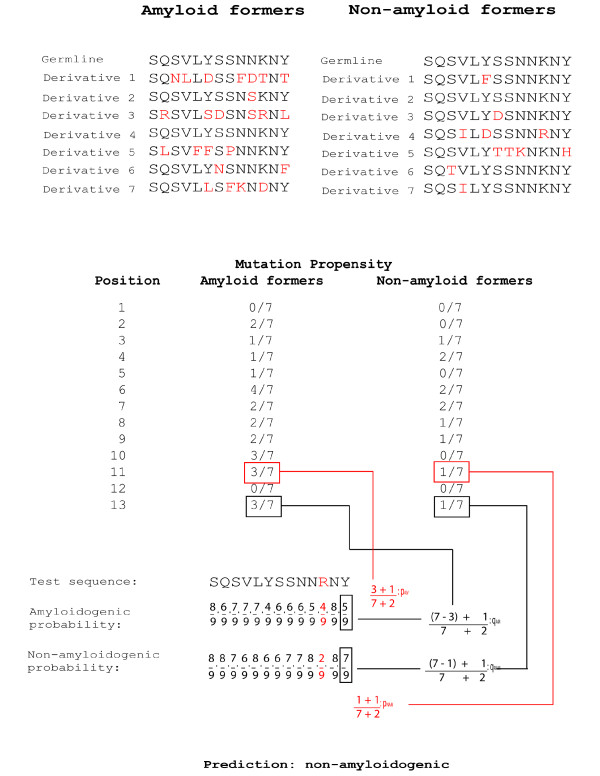
**Application of the naive Bayesian method for the prediction of amyloidosis**. Given a set of amyloidogenic and non-amyloidogenic derivatives of a single germline, it is possible to generate the probability that a mutation at a particular position would cause amyloidosis or not. Briefly, separate mutation propensities for amyloid (*p*_*AM*_) and non-amyloid (*p*_*NAM*_) formers are generated by counting the frequency of mutations per position. These fractions, as well as complements thereof (i.e. the probability that there will be no mutation in either an amyloid-former or non-amyloid-former at a particular position, in black) are subsequently used to compute the amyloidogenic and non-amyloidogenic probabilities of a test sequence. To calculate for the amyloidogenic probability of a test sequence, a probability is assigned to each of the n positions in the sequence based on the characteristic of that position (i.e. if it contains a mutation or not). For positions containing no mutations this probability is equivalent to *q*_*AM*_, *q*_*AM *_= 1 - *p*_*AM *_for position *x*. The probability for positions with mutations is equal to *p*_*AM *_. Non-amyloidogenic probabilities are calculated in a similar manner, but with the use of *p*_*NAM *_instead of *p*_*AM *_. To avoid multiplications by zero, the Laplace correction is used. A product of the probabilities is subsequently taken; if the product of amylodogenic probabilities is higher, the test sequence is classified as amyloidogenic.

This approach is attractive for the current problem, where there are only two possible outcomes. The most straightforward way of applying it is to use information of the combinations of positions at which mutations occur in amyloidogenic and non-amyloidogenic derivatives of a single germline. For example, to gauge the probability that a test sequence *x *derived from a germline *g *will be amyloidogenic, one would use the Bayes equation to evaluate the association between the positional combination of mutations, *c*, in *x *and the two hypotheses:(2)(3)

where *x*_*m*1_, *x*_*m*2_, ..., *x*_*mn *_define *c*, and with *p*_*AM *_and *p*_*NAM *_being defined by the positional mutational probabilities in amyloidogenic and non-amyloidogenic derivatives, respectively. Applying this method (Methods section, equations 4 and 5; Figure 4) yielded an average prediction accuracy of 60.8%; for an independent test set, the accuracy was 61.16% (Table 1). When the test set is used for training as well, the accuracy of amyloid sequence classification increases significantly. Misclassification of non-amyloidogenic sequences is also reduced by an average of 3% (Table 1, NAM Test). This correlation between the size of the training set and prediction accuracy has been previously observed [34]. It may be noteworthy to mention that the prediction accuracy for derivatives of the germline X72813 did not improve significantly even after the augmentation of the data set. Predictions for this germline are similarly low with the decision tree. Interestingly, most of the derivatives of X72813 are implicated in light chain deposition disease (LCDD). An interesting feature of LCDD-associated sequences is that when these are synthesized *in vitro*, the resulting proteins do not aggregate. Furthermore, the analysis of these sequences frequently show no obvious predisposition towards misfolding [35]. This may be a possible explanation for the difficulty in obtaining correct predictions for its amyloid-forming derivatives. If this set is treated as an outlier, the average prediction accuracy is 83.64 ± 18.49%.

In general, however, it is imperative to increase the training set size - not only in terms of the number of derivatives per germline, but in terms of the number of germlines covered, in order to improve the performance of the classifier. A development of a program for automatically generating training sets is a non-trivial task, however, and is beyond the scope of this study. It could also be possible to consider other characteristics, such as the physico-chemical and structural effects of a mutation, as factors for defining p_*AM *_or p_*NAM *_. Nevertheless, the question of how such factors would be incorporated in the calculation has to be justified first, from both statistical and biological points-of-view. Since our main interest is to provide a proof-of-concept that a simple set of classification algorithms may be used for predicting amyloidosis, we opted to complement the Bayesian method with a decision tree, where one could factor in additional effects of mutations for classifying sequences.

Decision trees are particularly useful in classifying unknowns into one of a finite number of categories, based on the results of a series of tests on the attributes of a sample [36,37]. It works by posing a series of questions about the features associated with unknowns; each question is contained in a node, and each node has child nodes for each possible answer to its question [38,39]. It eventually terminates in leaves, which correspond to a classification. There are many variants of decision trees; in the simplest form, 'yes'/'no' paths are followed throughout the classification process; in others, probability distributions over the classes are used in order to estimate the conditional probability that an item reaching a leaf belongs to the class if defines [39]. In biology, it has been used in Parkinson's disease management [40], disease severity profiling [41,42], toxicity analysis [43], large-scale proteomic studies [44,45], microarray data classification [46] and phylogenetic analysis, among other applications. Depending on the number of factors that will be considered to classify the samples, decision trees may be made by hand or constructed automatically using a learning or an optimization algorithm [38,47]. Choosing these factors and its arrangement on the tree to optimally separate samples remain challenges in the creation of decision trees; algorithms have since been developed for optimal tree creation [36-38]. For this study, four splitting variables were considered, based on the mutation trends observed in both amyloidogenic and non-amyloidogenic samples.

In order to obtain weights for the splitting variables, mutation matrices were generated for the amyloiodogenic and non-amyloidogenic derivatives of the different germlines. An interesting result from the analysis of these matrices is that 69% of the mutations exclusively found in exposed CDR residues of amyloid formers appear to be implicated in higher sheet-forming propensities, while 64% exclusive to buried FR residues involve shifts to residues with lower sheet-forming propensities (Figures 1 and 2, Table 2). This may suggest that mutations stabilizing sheet structures in the CDR, which normally assume loop structures, contribute as much to amyloidosis as those that destabilize the sheet structure in critical regions (i.e. buried FR residues). This is not unlikely, based on some previous observations. Hurle et al. [48], for instance, performed a positional analysis of 36 amyloidogenic sequences to find mutations that occur in less than 1% of all sequences at a particular position. These mutations were mostly found in CDRs, notably CDR1, for both *κ *and *λ *light chains. Furthermore, Stevens et al. observed that 24 out of the 26 invariant residues in *κ *light chains which drastically affect the structure of the antibody upon mutation are found on the protein surface, and make no obvious contributions to folding. Mutations in CDRs are generally more varied, and its contributions to amyloidosis, though not as easy to pinpoint, are probably very significant [49]. Finally, these results are consistent with predictions using other methods (see supplementary information, additional file 1); this consistency may be viewed as a validation of our observations.

From these observations, a decision tree was created to approximate the contribution of each mutation to the overall amyloidogenicity of a sequence. The use of this tree on the independent test set yielded a prediction accuracy of 78.64% (Table 6), which is close to the 75% prediction accuracy obtained when the decision tree is tested on training set sequences. LOO cross validation was not performed for this method, since this would require weights to be changed as many times as there are sequences. Classifiers generated with the training set appear to have a better performance than those from the naive Bayesian method. One possible reason was that more factors are taken into consideration - one approximates the effect of the mutation itself, as well as the effect that it has in being at a particular region; at the same time, it also roughly approximates the combined effect of mutations, which are likely to be equally responsible for misfolding as individual mutations [27,50]. Nevertheless, this does not imply that the naive Bayesian method is entirely without merit, since it is clear that position or combinations of positions where mutations occur has a key role in amyloidosis [27]. It is also evident that more sequences have to be used, as with the naive Bayesian method. Prediction results will also be probably improved by including additional factors such as hydrophilicity, size and charge changes as splitting variables, or refining the positions based on precedent studies [27]. In adding splitting variables, the construction of a decision tree could be performed using an [automated] optimization algorithm [38].

A caveat for both methods, however, is the possibility of overfitting, which is the description of random error, instead of true correlations. This phenomenon is one of the key problems in machine learning, and may occur when there are more degrees of freedom than data [51,52]. Overfitted model results are not representative of the population behavior, and are unlikely to be replicated. There are several rules of thumb for avoiding overfitting, which includes having a minimum of 10 - 15 observations per predictor variable, with larger sample sizes required in cases where the effect sizes are small, or when predictors are highly correlated [52]. For binary response models, the sample size may not be directly relevant [52], although for this problem, it appears that sample size plays an important role. Due to the limited sample set size, it was only possible to perform a single holdout validation and LOO cross validation, whose results were consistent. However, for future work involving larger training sets, it would be possible to include measures and perform more definitive tests to ensure that overfitting is eliminated or minimized.

## Conclusions

This exploratory study indicates that the Naive Bayesian classifier and decision trees may be used for "yes"- or "no"-type predictions on the amyloidogenicity of a sequence. Analysis of results from both methods suggests that prediction accuracy may be improved by optimizing the training set sizes, and by incorporating more information about the alterations brought about by mutations into the calculations. Some other factors that may be considered include hydrophilicity and charge changes brought about by the replacement residues, with respect to its location, as well as the way the mutations cluster from sequences with known structures. Another factor that might be considered is the sequence of immunoglobulin folding and the implications of having mutations in the N-terminal region, which is the first to be folded [53]. The further development of these classification techniques, including the possibility of creating a hybrid between Naive Bayesian and decision trees, appears to be worthwhile; these methods may eventually be adapted for predicting the amyloidogenicity of non-immunoglobulin sequences.

## Methods

### Sequences

The training set, comprised of 143 amyloidogenic and 158 non-amyloidogenic derivatives of the germlines were obtained from the National Center for Biotechnology Information (NCBI, http://www.ncbi.nlm.nih.gov/). A holdout test set comprised of 103 amyloidogenic and 28 non-amyloidogenic sequences, chosen on account of the absence of gaps, as well as the possibility of assigning these unambiguously to a germline set, were also obtained from the NCBI. Sequences were assigned to the closest germline using ClustalW, and resulting alignments were manually annotated. Kabat numbering and CDR/FR definitions were applied to all sequences. The non-amyloidogenic derivation sets were constructed from randomly chosen derivatives of each germline which have, as a derivation set, approximately the same total number of mutations as the amyloidogenic counterparts. The first five amino acid residues are omitted in the analysis, since these may have been primer-derived. All sequences of the amyloidogenic and non-amyloidogenic antibodies used in the analysis, which are identified by their NCBI accession codes, as well as their putative germline derivation, are in the supplementary information (additional file 2).

### Naive Bayesian Classification

We generated a Naive Bayesian Classifier for each germline on the basis of its amyloidogenic and non-amyloidogenic derivatives. Briefly, the probability *p *of a mutation occurring at position *x *was quantified for both amyloidogenic (*p*_*AM*_) and non-amyloidogenic (*p*_*NAM*_) derivatives of the same germline. Raw values of *p*_*AM *_and *p*_*NAM *_can take the value of 0; to avoid this, we used the Laplace correction method, where 1 is added to the numerator and 2 to the denominator. The respective complements, *q*_*AM *_and *q*_*NAM*_, which represent the retention of the residue, is given by 1 - *p*_*AM *_or 1 - *p*_*NAM*_, respectively. These probabilities are then used to calculate the amyloidogenic and non-amyloidogenic propensities for a test sequence *s *derived from the same germline as the training set. Supposing that *s *has mutations at positions defined by the set *M*, the amyloidogenic probability *AM *will be calculated as:(4)

while the non-amyloidogenic probability is calculated as:(5)

where x refers to the position (Figure 4). If AM is greater than NAM, then the sequence is classified as amyloidogenic; otherwise, it is classified as non-amyloidogenic. Classifier accuracy was cross-checked against both the training and test sets were used. Due to the limited number of sequences obtained, validation is preliminary, and consists of a LOO cross-validation, performed for all amyloidogenic and non-amyloidogenic derivatives, and a one-time holdout test validation.

### Decision tree generation and sequence classification

**Figure 5:**
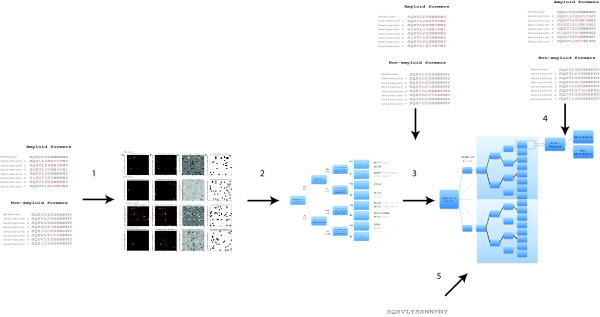
**Steps in generating and testing a weighted decision tree**. To create a weighted decision tree, mutations from amyloidogenic and non-amyloidogenic derivatives of a single germline are organized into separate matrices that factor in location, exposure and sheet-forming propensity into account (Step 1). These matrices are visualized and analyzed for general trends that may be transformed into weights (Step 2). An initial tree is constructed from these information, which is tested against the training set (Step 3). From this testing, it became evident that certain paths can be used for maximally separating amyloidogenic and non-amyloidogenic derivatives of a germline, and that these paths are germline-dependent. We then generated a tree that takes the germline of origin into account, and which has different boosted paths. The final step was to generate the classification threshold, which was determined from the analysis of scores for the test set (Step 4). This tree was then used to classify sequences in an independent, holdout test set (Step 5).

A weighted decision tree was constructed to provide a quantitative estimate of both individual and joint contributions of mutations as functions of location (i.e. CDR/FR), exposure and changes in sheet forming propensity. The steps for generating the tree are shown in Figure 5. Initially, separate mutation matrices for buried CDR residues, buried FR residues, exposed CDR residues, and exposed FR residues are generated for alignments of amyloidogenic and non-amyloidogenic derivatives, based on the algorithm described in [22]. Here, exposed residues were defined as residues having ≥ 25% accessible surface; exposure information was generated for each alignment using structural homologues of the germline sequence (see supplementary information, additional file 2). These were then visualized to facilitate easier analysis, then post-processed by subtracting the non-amyloidogenic from the amyloidogenic matrix image, resulting in an image where the relative intensities are proportional to the predominance of specific mutations. A binary matrix containing mutations exclusive to amyloid-formers was also generated. In the matrices, residues were arranged according to increasing *β*-sheet-forming propensities (Table 7) [54], with the original residues in the rows and the replacement residues in the columns, such that all mutations to the right of the diagonal are associated with increased sheet-forming propensities, while those to the left correspond to decreased sheet-forming propensities (Figure 2; Figure 5, step 1). The trends observed in these matrices (Figures 1, 2 and 5, step 2; Table 2) were then used as weights, which were associated with the branches of the tree. At this point, we determined if paths taken by amyloid and non-amyloid-formers could be generalized, or if these showed germline dependence. This led to the identification of paths that may be used in maximizing separation between amyloidogenic and non-amyloidogenic derivatives per germline (Table 4; Figure 5, step 3); for instance, amyloidogenic derivatives of X93627 can be maximally separated from corresponding non-amyloidogenic derivatives by giving a tenfold higher score to mutations that follow the path leading to leaf 2 and a tenfold lower score for mutations leading to leaf 8. Boosted and decreased paths to specific leaves are indicated in Table 4 in boldface and italics, respectively. Consequently, tracing the path through the tree that describes each mutation yields a score, *s*, calculated as the product of the weights along the path. Using this strategy, the average amyloidogenic potential for every sequence, *AM*_*seq*_, was calculated as follows:(6)

where *s *corresponds to scores of individual mutations, and *n *corresponds to the number of mutations in a sequence. Since *s *is amplified in certain paths, amyloidogenic sequences are expected to have higher *AM*_*seq *_values. Thresholds for classifying sequences as amyloidogenic or non-amyloidogenic were defined per germline based on the average scores of amyloidogenic derivatives (Figure 5, step 4). Cross-validation was performed on the holdout test set (Figure 5, step 5).

**Table 7:** *β*-sheet forming propensities of amino acids [54]

Amino acid	ΔΔ G (kcal mol^-1^)
Thr	1.1

Ile	1.0

Tyr	0.96

Phe	0.86

Val	0.82

Met	0.72

Ser	0.70

Trp	0.54

Cys	0.52

Leu	0.51

Arg	0.45

Lys	0.27

Gln	0.23

Glu	0.01

Ala	0.00

His	-0.02

Asn	-0.08

Asp	-0.94

Gly	-1.2

Pro	*<*-3

* N.A. indicates that no additional sequences were obtained for this germline.

## Authors' contributions

MPCD, GPC and EAP jointly conceptualized the project. EAP obtained and manually annotated the amyloidogenic sequences and their germline assignments. MPCD implemented the programs for Naive Bayesian analysis and decision tree-based classification and performed the analysis of the results. All authors have read and approved the manuscript in this form.

## Supplementary Material

Additional file 1 **Comparison of predictions between a germline and an amyloidogenic derivative made using AGGRESCAN **[**16**]** and the PASTA server **[**28**,**29**]. This shows that *regions *that may cause amyloidosis are predicted, with highly similar profiles. However, no direct predictions are provided (i.e. that the germline is non-amyloidogenic, and that the derivative is amyloidogenic) in these methods. Click here for file

Additional file 2 **Amyloidogenic and non-amyloidogenic immunoglobulin sequence alignments for each of the germline derivation sets, including the exposure data.** The structure indicated at the end of each alignment refers to the structural template used as the basis for determining residue exposure. Sequences in red are those belonging to the holdout test set. Click here for file

## References

[B1] Presta L Antibody engineering Curr Opin Biotechnol 1992 3 394 398 10.1016/0958-1669(92)90168-I 1368441

[B2] Presta L Antibody engineering for therapeutics Current Opinion in Structural Biology 2003 13 4 519 525 10.1016/S0959-440X(03)00103-9 12948783

[B3] Padlan E A possible procedure for reducing the immunogenicity of antibody variable domains while preserving their ligand-binding properties Molecular Immunology 1991 28 4-5 489 498 10.1016/0161-5890(91)90163-E 1905784

[B4] Roguska M Pedersen J Keddy C Humanization of murine monoclonal antibodies through variable domain resurfacing Proceedings of the National Academy of Sciences 1994 91 969 973 10.1073/pnas.91.3.969 PMC521435 8302875

[B5] Clark M Antibody humanization: a case of the 'Emperor's new clothes'? Immunol Today 2000 21 397 402 10.1016/S0167-5699(00)01680-7 10916143

[B6] Ewert S Honegger A Plückthun A Stability improvement of antibodies for extracellular and intracellular applications: CDR grafting to stable frameworks and structure-based framework engineering Methods 2004 34 2 184 199 10.1016/j.ymeth.2004.04.007 15312672

[B7] Hurle M Helms L Li L Chan W Wetzel R A role for destabilizing amino acid replacements in light-chain amyloidosis Proceedings of the National Academy of Sciences 1994 91 5446 5450 10.1073/pnas.91.12.5446 PMC44012 8202506

[B8] Mateo C Humanization of a mouse monoclonal antibody that blocks the epidermal growth factor receptor: recovery of antagonistic activity Immunotechnology 1997 3 71 81 10.1016/S1380-2933(97)00065-1 9154469

[B9] de la Paz ML Serrano L Sequence determinants of amyloid fibril formation Proceedings of the National Academy of Sciences 2004 101 87 92 10.1073/pnas.2634884100 PMC314143 14691246

[B10] Srisailam S Wang HM Kumar T Rajalingam D Sivaraja V Sheu HS Chang YC Yu C Amyloid-like Fibril Formation in an All beta-Barrel Protein Involves the Formation of Partially Structured Intermediate(s) Journal of Biological Chemistry 2002 277 21 19027 10.1074/jbc.M110762200 11877422

[B11] Villegas V Zurdo J Filimonov V Aviles F Dobson C Serrano L Protein engineering as a strategy to avoid formation of amyloid fibrils Protein Science 2000 9 1700 1708 10.1110/ps.9.9.1700 11045616 PMC2144697

[B12] Vidal R Goni F Stevens F Aucouturier P Kumar A Frangione B Ghiso J Gallo G Somatic Mutations of the L12a Gene in V-kappa1 Light Chain Deposition Disease: Potential Effects on Aberrant Protein Conformation andDeposition American Journal of Pathology 1999 155 6 2009 10595931 10.1016/s0002-9440(10)65520-4 PMC1866929

[B13] Uversky VN Fink AL Conformational constraints for amyloid fibrillation: the importance of being unfolded Biochimica et Biophysica Acta (BBA) - Proteins & Proteomics 2004 1698 2 131 153 10.1016/j.bbapap.2003.12.008 15134647

[B14] Ding F Borreguero J Buldyrey S Mechanism for the-helix to-hairpin transition Proteins: Structure, Function and Genetics 2003 53 220 228 10.1002/prot.10468 14517973

[B15] Gross M Gross M Wilkins DK Wilkins DK Pitkeathly MC Pitkeathly MC Chung EW Chung EW Higham C Higham C Clark A Clark A Dobson CM Dobson CM Formation of amyloid fibrils by peptides derived from the bacterial cold shock protein CspB Protein Sci 1999 8 6 1350 10.1110/ps.8.6.1350 10386885 PMC2144354

[B16] Conchillo-Solé O Groot NSD Avilés FX Vendrell J Daura X Ventura S AGGRESCAN: a server for the prediction and evaluation of "hot spots" of aggregation in polypeptides BMC bioinformatics 2007 8 65 10.1186/1471-2105-8-65 17324296 PMC1828741

[B17] Caflisch A Computational models for the prediction of polypeptide aggregation propensity Current opinion in chemical biology 2006 10 5 437 44 10.1016/j.cbpa.2006.07.009 16880001

[B18] Zavaljevski N Stevens F Reifman J Support vector machines with selective kernel scaling for protein classification and identification of key amino acid positions Bioinformatics 2002 18 689 696 10.1093/bioinformatics/18.5.689 12050065

[B19] Galzitskaya O Garbuzynskiy S Lobanov M Prediction of amyloidogenic and disordered regions in protein chains PLoS Comput Biol 2006 2 e177 10.1371/journal.pcbi.0020177 17196033 PMC1761655

[B20] Behar SM Scharff MD Somatic diversification of the S107 (T15) VH11 germ-line gene that encodes the heavy-chain variable region of antibodies to double-stranded DNA in (NZB × NZW)F1 mice Proc Natl Acad Sci USA 1988 85 11 3970 10.1073/pnas.85.11.3970 3131767 PMC280342

[B21] Hawkins D The problem of overfitting J Chem Inf Comput Sci 2004 44 1 12 14741005 10.1021/ci0342472

[B22] David M Asprer J Ibana J Concepcion G Padlan E A study of the structural correlates of affinity maturation: antibody affinity as a function of chemical interactions, structural plasticity and stability Molecular Immunology 2007 44 1342 1351 10.1016/j.molimm.2006.05.006 16854467

[B23] David M Lapid C Daria V An efficient visualization tool for the analysis of protein mutation matrices BMC bioinformatics 2008 9 218 10.1186/1471-2105-9-218 18442400 PMC2390542

[B24] Stevens FJ Argon Y Pathogenic light chains and the B-cell repertoire Immunol Today 1999 20 10 451 7 10.1016/S0167-5699(99)01502-9 10500292

[B25] Perfetti V Ubbiali P Vignarelli M Diegoli M Fasani R Stoppini M Lisa A Mangione P Obici L Arbustini E Evidence that amyloidogenic light chains undergo antigen-driven selection Blood 1998 91 8 2948 9531605

[B26] Stefani M Protein misfolding and aggregation: new examples in medicine and biology of the dark side of the protein world BBA-Molecular Basis of Disease 2004 1739 5 25 10.1016/j.bbadis.2004.08.004 15607113

[B27] Poshusta TL Sikkink LA Leung N Clark RJ Dispenzieri A Ramirez-Alvarado M Hofmann A Mutations in Specific Structural Regions of Immunoglobulin Light Chains Are Associated with Free Light Chain Levels in Patients with AL Amyloidosis PLoS ONE 2009 4 4 e5169 10.1371/journal.pone.0005169 19365555 PMC2664898

[B28] Trovato A Seno F Tosatto S The PASTA server for protein aggregation prediction Protein Engineering Design and Selection 2007 20 521 523 10.1093/protein/gzm042 17720750

[B29] Trovato A Chiti F Maritan A Seno F Insight into the structure of amyloid fibrils from the analysis of globular proteins PLoS Comput Biol 2006 2 1608 1618 10.1371/journal.pcbi.0020170 PMC1698942 17173479

[B30] Zhang Z Chen H Lai L Identification of amyloid fibril-forming segments based on structure and residue-based statistical potential Bioinformatics 2007 23 17 2218 2225 10.1093/bioinformatics/btm325 17599928

[B31] Tartaglia GG Pawar AP Campioni S Dobson CM Chiti F Vendruscolo M Prediction of aggregation-prone regions in structured proteins J Mol Biol 2008 380 2 425 36 10.1016/j.jmb.2008.05.013 18514226

[B32] Tian J Wu N Guo J Fan Y Prediction of amyloid fibril-forming segments based on a support vector machine BMC bioinformatics 2009 10 Suppl 1 S45 10.1186/1471-2105-10-S1-S45 19208147 PMC2648769

[B33] Mitchell T Machine Learning 1997 McGraw Hill

[B34] Vega V Bressan S et al TS Continuous Naive Bayesian classifications Lecture Notes in Computer Science 2003 2911 Heidelberg: Springer 279 289

[B35] Rocca A Khamlichi A Aucouturier P Noel L Denoroy L Preud'homme J Cogne M Primary structure of a variable region of the V kappa I subgroup (ISE) in light chain deposition disease Clinical and Experimental Immunology 1993 91 506 509 7680298 10.1111/j.1365-2249.1993.tb05932.x PMC1554718

[B36] Moret B Decision trees and diagrams Computing Surveys 1982 4 595 623

[B37] Quinlan J Decision trees and decision-making IEEE transactions on systems, man and cybernetics 1990 20 339 346 10.1109/21.52545

[B38] Norton S Sridharan N Generating better decision trees Proceedings of the Eleventh International Joint Conference on Artificial Intelligence, Detroit, MI, USA 1989 800805 800 805

[B39] Kingsford C Salzberg SL What are decision trees? Nat Biotechnol 2008 26 9 1011 10.1038/nbt0908-1011 18779814 PMC2701298

[B40] Olanow C Watts R Koller W An algorithm (decision tree) for the management of Parkinson's disease (2001): treatment guidelines Neurology 2001 56 1 88 10.1212/wnl.56.suppl_5.s1 11402154

[B41] Adam B Qu Y Davis J Ward M Clements M Cazares L Semmes O Schellhammer P Yasui Y Feng Z Wright G Serum Protein Fingerprinting Coupled with a Pattern-matching Algorithm Distinguishes Prostate Cancer from Benign Prostate Hyperplase and Healthy Men Cancer Research 2002 62 3609 3614 12097261

[B42] Kang X Xu Y Wu X Liang Y Wang C Guo J Proteomic Fingerprints for Potential Application to Early Diagnosis of Severe Acute Respiratory Syndrome Clinical Chemistry 2005 51 56 64 10.1373/clinchem.2004.032458 15550479 PMC7108460

[B43] Dunkley E Isbister G Sibbritt D The Hunter Serotonin Toxicity Criteria: simple and accurate diagnostic decision rules for serotonin toxicity Q J Med 2003 96 635 642 10.1093/qjmed/hcg109 12925718

[B44] Christendat D Yee A Dharamsi A Kluger Y Savchenko A Cort JR Booth V Mackereth CD Saridakis V Ekiel I Kozlov G Maxwell KL Wu N Mcintosh LP Gehring K Kennedy MA Davidson AR Pai EF Gerstein M Edwards AM Arrowsmith CH Structural proteomics of an archaeon Nature Structural & Molecular Biology 2000 7 10 903 10.1038/82823 11017201

[B45] Geurts P Fillet M Seny DD Meuwis M Proteomic mass spectra classification using decision tree based ensemble methods Bioinformatics 2005 21 318 3145 10.1093/bioinformatics/bti494 15890743

[B46] Wang Y Tetko I Hall M Frank E Gene selection from microarray data for cancer classification--a machine learning approach Computational Biology and Chemistry 2005 29 37 46 10.1016/j.compbiolchem.2004.11.001 15680584

[B47] Bennett K Evans M Decision tree construction via linear programming Proceedings of the 4th Midwest Artificial Intelligence and Cognitive Science Society Conference, Utica, Illinois 1992 97 101

[B48] Hurle M Helms L Li L Chan W Wetzel R A role for destabilizing amino acid replacements in light-chain amyloidosis Proceedings of the National Academy of Sciences 1994 91 12 5446 5450 10.1073/pnas.91.12.5446 PMC44012 8202506

[B49] Abraham RS Geyer SM Ramírez-Alvarado M Price-Troska TL Gertz MA Fonseca R Analysis of somatic hypermutation and antigenic selection in the clonal B cell in immunoglobulin light chain amyloidosis (AL) J Clin Immunol 2004 24 4 340 53 10.1023/B:JOCI.0000029113.68758.9f 15163890

[B50] Depristo MA Weinreich DM Hartl DL Missense meanderings in sequence space: a biophysical view of protein evolution Nature Reviews Genetics 2005 6 9 678 687 10.1038/nrg1672 16074985

[B51] Vezhnevets A Barinova O et al K Avoiding boosting overfitting by removing confusing samples European Conference on Machine Learning (ECML07), LNAI 2007 430 441

[B52] Babyak M What you see may not be what you get: a brief, nontechnical introduction to overfitting in regression-type models Psychosomatic Medicine 2004 66 411 421 10.1097/01.psy.0000127692.23278.a9 15184705

[B53] Zanetti M Capra J The antibodies 1996 1 CRC Press

[B54] Minor DL Kim PS Measurement of the beta-sheet-forming propensities of amino acids Nature 1994 367 6464 660 3 10.1038/367660a0 8107853

