# Supplementary material for: Using simple artificial intelligence methods for predicting amyloidogenesis in antibodies
Source: BMC Bioinformatics. 2010 Feb 8;11:79. doi: 10.1186/1471-2105-11-79 (PMC3098112; doi:10.1186/1471-2105-11-79)
Supplement: Additional file 1 — Comparison of predictions between a germline and an amyloidogenic derivative made using AGGRESCAN [16] and the PASTA server [28,29]. This shows that regions that may cause amyloidosis are predicted, with highly similar profiles. However, no direct predictions are provided (i.e. that the germline is non-amyloidogenic, and that the derivative is amyloidogenic) in these methods. [file 1471-2105-11-79-S1.PDF]

		Average over all sequences
Sequence Name:	J00248	
Graphics:		
a3v Sequence Average (a3vSA):	-0.022	-0.022
Number of Hot Spots (nHS):	4	4.000
Normalized nHS for 100 residues (NnHS):	4.211	4.211
Area of the profile Above Threshold (AAT):	11.540	11.540
Total Hot Spot Area (THSA):	9.636	9.636
Total Area (TA):	-1.230	-1.230
AAT per residue (AATr):	0.121	0.121
THSA per residue (THSAr):	0.101	0.101
Normalized a4v Sequence Sum for 100 residues (Na4vSS):	-3.7	-3.700

#	AA	a4v	HSA	NHSA	a4vAHS
1	D	-0.478	0.000	0.000	0.000
2	I	-0.478	0.000	0.000	0.000
3	Q	-0.478	0.000	0.000	0.000
4	M	-0.288	0.000	0.000	0.000
5	T	-0.074	0.000	0.000	0.000
6	Q	-0.376	0.000	0.000	0.000
7	S	-0.242	0.000	0.000	0.000
8	P	-0.175	0.000	0.000	0.000
9	S	-0.194	0.000	0.000	0.000
10	S	-0.024	0.000	0.000	0.000
11	L	-0.024	0.000	0.000	0.000
12	S	0.252	0.526	0.000	0.155
13	A	0.217	0.526	0.000	0.155
14	S	-0.003	0.526	0.000	0.155
15	V	-0.377	0.000	0.000	0.000
16	G	-0.108	0.000	0.000	0.000
17	D	-0.125	0.000	0.000	0.000
18	R	0.177	0.197	0.000	0.177
19	V	-0.073	0.000	0.000	0.000
20	T	0.089	0.767	0.000	0.172
21	I	0.175	0.767	0.000	0.172
22	T	0.347	0.767	0.000	0.172
23	C	0.077	0.767	0.000	0.172
24	R	-0.076	0.000	0.000	0.000
25	A	-0.413	0.000	0.000	0.000
26	S	-0.130	0.000	0.000	0.000
27	Q	-0.258	0.000	0.000	0.000
28	G	-0.267	0.000	0.000	0.000
29	I	-0.096	0.000	0.000	0.000
30	S	0.143	2.567	0.367	0.347
31	N	0.313	2.567	0.367	0.347
32	Y	0.538	2.567	0.367	0.347
33	L	0.528	2.567	0.367	0.347
34	A	0.394	2.567	0.367	0.347
35	W	0.405	2.567	0.367	0.347

Sorted by Na4vSS

J00248	-3.70

36	F	0.106	2.567	0.367	0.347
37	Q	-0.139	0.000	0.000	0.000
38	Q	-0.210	0.000	0.000	0.000
39	K	-0.491	0.000	0.000	0.000
40	P	-0.747	0.000	0.000	0.000
41	G	-0.619	0.000	0.000	0.000
42	K	-0.576	0.000	0.000	0.000
43	A	-0.485	0.000	0.000	0.000
44	P	-0.240	0.000	0.000	0.000
45	K	0.097	3.163	0.395	0.375
46	S	0.395	3.163	0.395	0.375
47	L	0.395	3.163	0.395	0.375
48	I	0.438	3.163	0.395	0.375
49	Y	0.529	3.163	0.395	0.375
50	A	0.529	3.163	0.395	0.375
51	A	0.529	3.163	0.395	0.375
52	S	0.093	3.163	0.395	0.375
53	S	-0.115	0.000	0.000	0.000
54	L	-0.186	0.000	0.000	0.000
55	Q	0.047	0.188	0.000	0.043
56	S	0.041	0.188	0.000	0.043
57	G	0.041	0.188	0.000	0.043
58	V	-0.333	0.000	0.000	0.000
59	P	0.093	0.000	0.000	0.000
60	S	0.093	0.226	0.000	0.093
61	R	0.093	0.226	0.000	0.093
62	F	-0.177	0.000	0.000	0.000
63	S	-0.205	0.000	0.000	0.000
64	G	-0.205	0.000	0.000	0.000
65	S	-0.105	0.000	0.000	0.000
66	G	-0.378	0.000	0.000	0.000
67	S	-0.598	0.000	0.000	0.000
68	G	-0.271	0.000	0.000	0.000
69	T	-0.252	0.000	0.000	0.000
70	D	0.022	2.561	0.320	0.300
71	F	0.041	2.561	0.320	0.300
72	T	0.378	2.561	0.320	0.300
73	L	0.358	2.561	0.320	0.300
74	T	0.579	2.561	0.320	0.300
75	I	0.525	2.561	0.320	0.300
76	S	0.372	2.561	0.320	0.300
77	S	0.127	2.561	0.320	0.300
78	L	-0.052	0.000	0.000	0.000
79	Q	-0.574	0.000	0.000	0.000
80	P	-0.282	0.000	0.000	0.000
81	E	-0.245	0.000	0.000	0.000
82	D	-0.465	0.000	0.000	0.000
83	F	-0.123	0.000	0.000	0.000
84	A	0.090	1.344	0.224	0.204
85	T	0.378	1.344	0.224	0.204
86	Y	0.464	1.344	0.224	0.204
87	Y	0.038	1.344	0.224	0.204
88	C	0.209	1.344	0.224	0.204
89	Q	0.045	1.344	0.224	0.204
90	Q	-0.162	0.000	0.000	0.000
91	Y	-0.162	0.000	0.000	0.000
92	N	-0.296	0.000	0.000	0.000
93	S	-0.275	0.000	0.000	0.000
94	Y	-0.275	0.000	0.000	0.000
95	P	-0.275	0.000	0.000	0.000

	Sequence Name: AAC97098	Average over all sequences
Graphics:		
a3v Sequence Average (a3vSA):	-0.016	-0.016
Number of Hot Spots (nHS):	4	4.000
Normalized nHS for 100 residues (NnHS):	4.211	4.211
Area of the profile Above Threshold (AAT):	11.084	11.084
Total Hot Spot Area (THSA):	8.755	8.755
Total Area (TA):	-1.277	-1.277
AAT per residue (AATr):	0.117	0.117
THSA per residue (THSAr):	0.092	0.092
Normalized a4v Sequence Sum for 100 residues (Na4vSS):	-3.5	-3.500

#	AA	a4v	HSA	NHSA	a4vAHS
1	D	-0.075	0.000	0.000	0.000
2	I	-0.075	0.000	0.000	0.000
3	V	-0.075	0.000	0.000	0.000
4	M	0.115	0.532	0.000	0.157
5	T	0.330	0.532	0.000	0.157
6	Q	0.027	0.532	0.000	0.157
7	S	-0.242	0.000	0.000	0.000
8	P	-0.175	0.000	0.000	0.000
9	S	-0.194	0.000	0.000	0.000
10	S	-0.024	0.000	0.000	0.000
11	L	-0.024	0.000	0.000	0.000
12	S	0.252	0.526	0.000	0.155
13	A	0.217	0.526	0.000	0.155
14	S	-0.003	0.526	0.000	0.155
15	V	-0.377	0.000	0.000	0.000
16	G	-0.108	0.000	0.000	0.000
17	D	-0.125	0.000	0.000	0.000
18	R	0.177	0.197	0.000	0.177
19	V	-0.073	0.000	0.000	0.000
20	T	0.089	0.767	0.000	0.172
21	I	0.175	0.767	0.000	0.172
22	T	0.347	0.767	0.000	0.172
23	C	0.077	0.767	0.000	0.172
24	R	-0.076	0.000	0.000	0.000
25	A	-0.599	0.000	0.000	0.000
26	S	-0.316	0.000	0.000	0.000
27	Q	-0.444	0.000	0.000	0.000
28	D	-0.453	0.000	0.000	0.000
29	I	-0.282	0.000	0.000	0.000
30	S	-0.043	0.000	0.000	0.000
31	N	0.128	2.219	0.370	0.350
32	Y	0.538	2.219	0.370	0.350
33	L	0.528	2.219	0.370	0.350
34	A	0.394	2.219	0.370	0.350
35	W	0.405	2.219	0.370	0.350

Sorted by Na4vSS

AAC97098 -3.50

36	F	0.106	2.219	0.370	0.350
37	Q	-0.139	0.000	0.000	0.000
38	Q	-0.210	0.000	0.000	0.000
39	K	-0.491	0.000	0.000	0.000
40	P	-0.747	0.000	0.000	0.000
41	G	-0.619	0.000	0.000	0.000
42	K	-0.576	0.000	0.000	0.000
43	A	-0.485	0.000	0.000	0.000
44	P	-0.240	0.000	0.000	0.000
45	K	0.097	2.578	0.368	0.348
46	S	0.395	2.578	0.368	0.348
47	L	0.358	2.578	0.368	0.348
48	I	0.401	2.578	0.368	0.348
49	Y	0.492	2.578	0.368	0.348
50	S	0.348	2.578	0.368	0.348
51	A	0.348	2.578	0.368	0.348
52	S	-0.088	0.000	0.000	0.000
53	N	-0.259	0.000	0.000	0.000
54	L	-0.293	0.000	0.000	0.000
55	Q	-0.061	0.000	0.000	0.000
56	A	-0.066	0.000	0.000	0.000
57	G	0.078	0.098	0.000	0.078
58	V	-0.305	0.000	0.000	0.000
59	P	0.121	0.000	0.000	0.000
60	S	0.084	0.208	0.000	0.084
61	N	0.084	0.208	0.000	0.084
62	F	-0.220	0.000	0.000	0.000
63	S	-0.249	0.000	0.000	0.000
64	G	-0.249	0.000	0.000	0.000
65	G	-0.139	0.000	0.000	0.000
66	G	-0.412	0.000	0.000	0.000
67	S	-0.633	0.000	0.000	0.000
68	G	-0.306	0.000	0.000	0.000
69	T	-0.252	0.000	0.000	0.000
70	D	0.022	2.561	0.320	0.300
71	F	0.041	2.561	0.320	0.300
72	T	0.378	2.561	0.320	0.300
73	L	0.358	2.561	0.320	0.300
74	T	0.579	2.561	0.320	0.300
75	I	0.525	2.561	0.320	0.300
76	S	0.372	2.561	0.320	0.300
77	S	0.127	2.561	0.320	0.300
78	L	-0.052	0.000	0.000	0.000
79	Q	-0.574	0.000	0.000	0.000
80	P	-0.282	0.000	0.000	0.000
81	E	-0.245	0.000	0.000	0.000
82	D	-0.465	0.000	0.000	0.000
83	F	-0.123	0.000	0.000	0.000
84	A	0.090	1.397	0.233	0.213
85	T	0.378	1.397	0.233	0.213
86	Y	0.464	1.397	0.233	0.213
87	Y	0.038	1.397	0.233	0.213
88	C	0.209	1.397	0.233	0.213
89	Q	0.098	1.397	0.233	0.213
90	Q	-0.109	0.000	0.000	0.000
91	Y	-0.109	0.000	0.000	0.000
92	K	-0.243	0.000	0.000	0.000
93	S	-0.222	0.000	0.000	0.000
94	Y	-0.222	0.000	0.000	0.000
95	P	-0.222	0.000	0.000	0.000

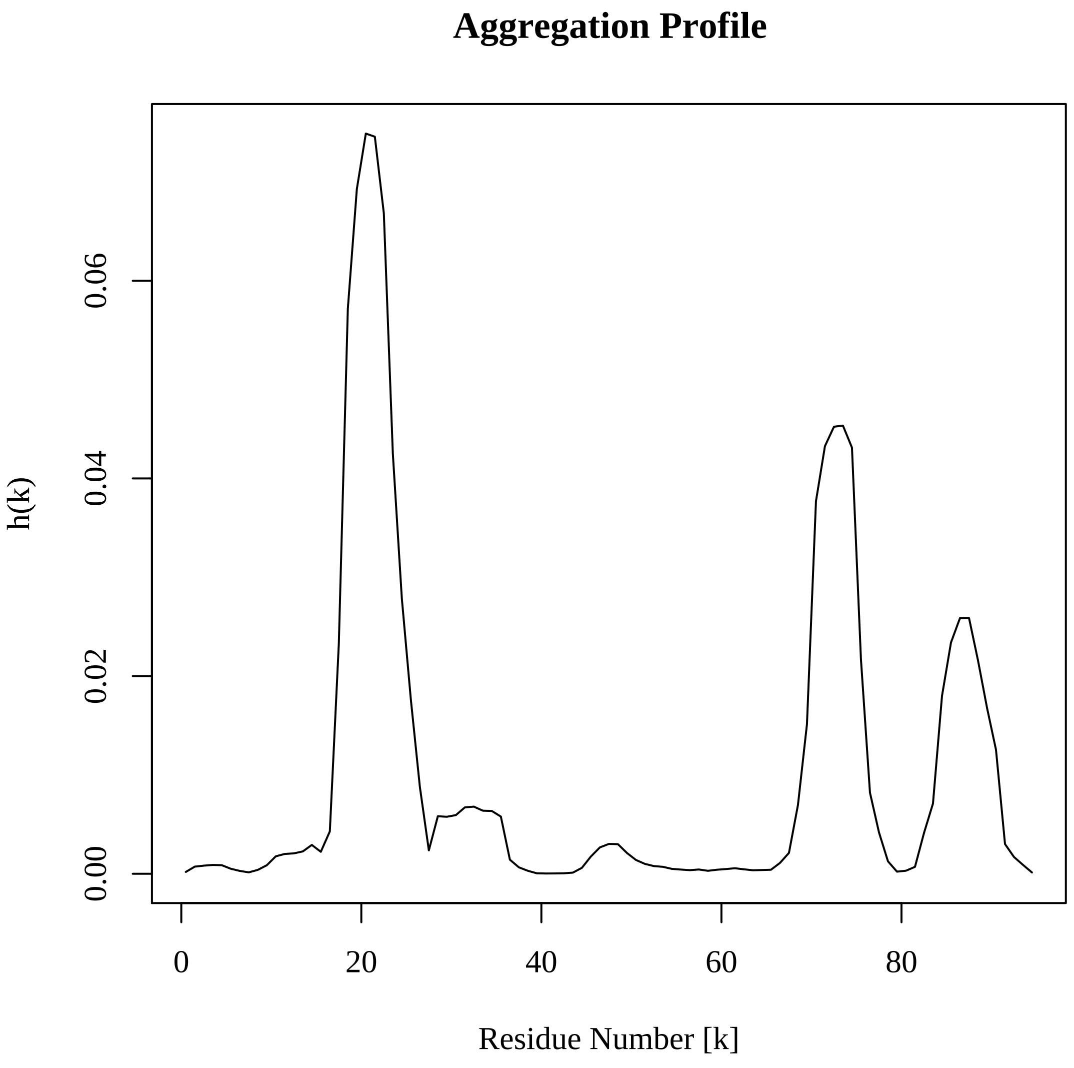

Aggregation Profile
h(k)
0.00
0.02
0.04
0.06
0
20
40
60
80
Residue Number [k]

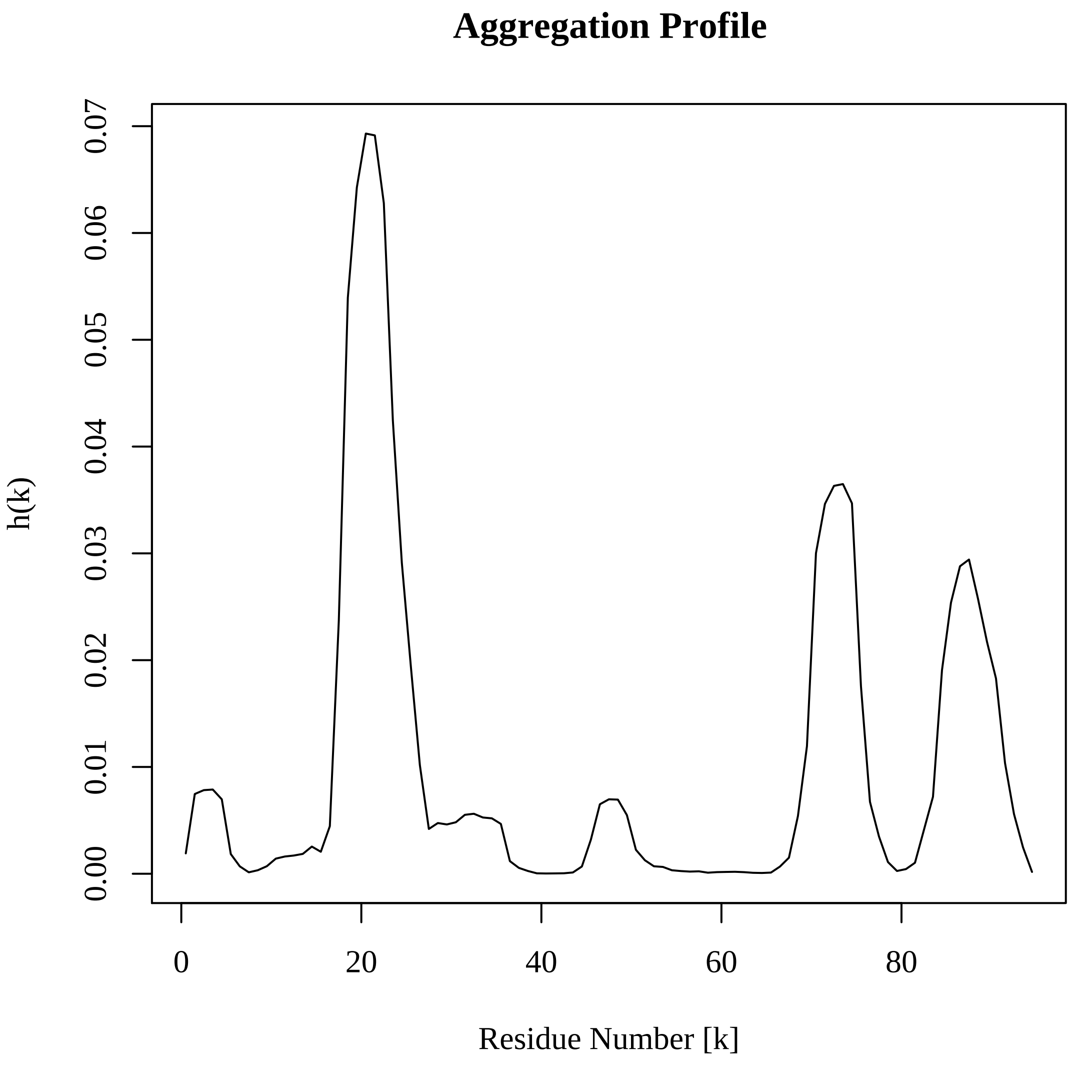
Aggregation Profile
h(k)
0.00
0.01
0.02
0.03
0.04
0.05
0.06
0.07
0
20
40
60
80
Residue Number [k]
