# Supplementary material for: Using simple artificial intelligence methods for predicting amyloidogenesis in antibodies
Source: BMC Bioinformatics. 2010 Feb 8;11:79. doi: 10.1186/1471-2105-11-79 (PMC3098112; doi:10.1186/1471-2105-11-79)
Supplement: Additional file 2 — Amyloidogenic and non-amyloidogenic immunoglobulin sequence alignments for each of the germline derivation sets, including the exposure data. The structure indicated at the end of each alignment refers to the structural template used as the basis for determining residue exposure. Sequences in red are those belonging to the holdout test set. [file 1471-2105-11-79-S2.PDF]

Amyloidogenic sequences

```
J00248 (L1)  DIQMTQSPSSLSASVGDRVTITCRASQGISNYLAWFQQKPGKAPKSLIYAASSLQSGVPSRFSGSGSGTDFTLTISSLQPEDFATYYCQQYNSYP
AAC97098     DIVMTQSPSSLSASVGDRVTITCRASQDISNYLAWFQQKPGKAPKSLIYSASNLQAGVPSNFSGGGSGTDFTLTISSLQPEDFATYYCQQYKSYP
AAC97099     DIVMTQSPSSLSASVGDRVTITCRASQDIKNFLAWLQQKPGKAPKSLIYSASSLQSGVPSNFSGGGSGTHFTLTITSLQPEDFATYYCQQYDSFP
AAC97100     DIEMTQSPSSLSASVGDRVTITCRASQDISNFLAWFQQKPGNAPKSLIYAASSLQSGVPSKFSGGGSGTNFTLTISSLQPEDFATYYCQQYNNYP
AAC97101     DIVMTQSPSSLSASIGDRVSITCRASQGISNFLAWFQQKPGKAPKSLIYAASSLQSGVSSNFSGSGSGTDFTLTISSLQPEDFATYYCQQYNSYP
AAD09377     DIQMTQSPSSVSASVGDRLTISCRASQDISTYLAWFQQKPGKAPKSLIYAASNLQSGVPSNFSGSGSGIEFTLTINSLQPEDFATYYCQQYNIYP
BAN          DIQLTQSPSSLSASVGDRVTITCRASQSVYNYVAWFQQKPGKAPKSLIYDASTLQSGVPSNFTGSGSGTDFILTISSLQPEDFATYYCQQYNSYP
HAC          DIQMTQSPPSVSASVGDRVTITCRASQGISNYLAWFQQKPGKAPKSLIYAASSLQSGVPSKFSGSGSGTDFTLTISSLQPEDFATYYCQQYSSSP
NIE          DIQMTQSPSSLPASVGDRVIITCRASQAIGNYLAWFQQKPGKAPKSLIYAASNLQSGVSSKFSGSGLGTDFTLTISSLQPEDFATYYCQQYNSNP
             ebebebebeebebbebeebebbbebeeebbbebbbbbbbeeebbebbbbbbeeebeebbebbbbebebbebbbbbbbbbeebbbbbbbbbeeeb
Structure: 1B6D

X72813       DIQMTQSPSTLSASVGDRVTITCRASQSISSWLAWYQQKPGKAPKLLIYKASSLESGVPSRFSGSGSGTEFTLTISSLQPDDFATYYCQQYNSYS
COL          DIQMTQSPSTLSASVGDRVTITCRASQTIINWLAWYQQKPGEAPKLLIYKASTLQSGVPSRFSGSGSGTEFTLTISSLQPEDVATYYCQQYNSAP
HOWE         DIQMTQSPSTLSASVGDRVTITCRASQTISTSLAWYQQKPGNAPKLLIYRASSLQSGVPSTFSGSGSGTEFTLTISSLQPDDFATYYCQHYRTYP
A49134 (LCDD)DIQMTQSPSTLSASVGDRVTITCRASLGINIWLAWYQQKPGKAPNLLIYKATNLQSGVPSRFSASGSGTEFTLTISGLQPDDFATYYCHQDNSYP
LW           DIQMTQSPSTLSASVGDRVTITCRASQSITNYLAWYQQKAGKAPSLLIYKASTLETGVPSRFSGGGSGTHFTLNISSLQPDDFATYYCQQYNTYP
MCM (LCDD)   DIQMTQSPSTLSASVGDRVTITCRASQSIGNWNAWYQEKPGQAPKLLIYKASTLESGVPSRFSGEGSGTEFTLTISKLQPDIFATYYCQQIFSLP
WIL2 (MCN)   DIQMTQSPSTLSASVGDRVTITCRPSQAFGSWLAWYQQKPGKAPELLIYRVSSLQSGVPSRFSGSGSGTEFTLTISSLQPDDVATYYCQQANRYS
ABU90582     DIQMTQSPSTLSASVGDRVTITCRASQSISRWLAWYQQKPGNAPNLLIYKASTLERGVPSRFSGSGSGTEFTLTISSLQPDDFATYYCQEYSSDW
ABU90709     DIQMTQSPSSLSASVGDRVTITCRASESINEYLAWYQQKPAKAPTLLIFKASTLERGAASRFSGSGSGTEFTLTISSLQPDDFATYYCQQYYSSY
             ebebebbbeebbbbebbebebbbebeeebbebbbbbbbbeebbbebbbbbbebbbeebbebbbbebebeebbbbbebbeeebbbbbbbbbebbb
Structure: 1DFB


                      .         .          .         .         .
X93620       DIQMTQSPSSLSASVGDRVTITCQASQDISNYLNWYQQKPGKAPKLLIYDASNLETGVPSRFSGSGSGTDFTFTISSLQPEDIATYYCQQYDNLP
AAA79238     DIQMTQSPSSLSASVGDRVTITCQASQDISDYLIWYQQKLGKAPNLLIYDASTLETGVPSRFSGSGSGTEYTFTISSLQPEDIATYYCQQYDDLP
AAC97102     DIQMTQSPSSLSASVGDRVTITCQASQDINNYLNWYQQKPGKTPKLLIYGASNLETGVPSRFSGSGSGTDFIFTISSLQPEDIATYYCQQYDNLP
AAC97103     DIVMTQSPSSLSASVGDRVTITCQASQDISNYLNWYQQKPGKAPKLLIDGASNLETGVPSRFSGSGSGTDFTFTISSLQPEDIATYYCQQYDNLP
AAD09367     DIQMTQSPSSLSASVGDRVTITCQASQDIHNYLNWYQQKPGKAPKLLIYDASKLETGVPSKFSGNGSGTGFTFTISSLQSEDIATYYCQQYNDLP
AAD29304     DIVMTQSPSSLSASVGDRVTITCQASQDIANYLNWYQQRPGKAPKVLIYDASNLEIGVPSRFSGTGSGTHFNFTISGLQPEDIAVYSCQQYDNLP
AAD29303     DIEMTQSPSSLSASVGNRVIITCQASQDITNFLNWYQQKPGKAPKLLIYDASNLQKGVPSTFSGSGSGTDFTFTISSLQPEDIATYYCQQYANLV
AM81         DIQMTQSPSTLSASVGDRVIITCRASQSVLTYLNWYQQKPGKAPKLIIYDATLLLTGVPSKFSGSGSGSDFNISISGLQPEDFATYYCQQFDAGP
ARN          DIQMTQSPSSLSASVGDGVTLTCQASQDISDYLNWYQQKVGEAPKLLMYDASYLETGVPLRFSGSGSGTNYSFTISSLQPEDFATYYCQQYSNLP
BRE          DIQMTQSPSSLSASVGDRVTITCQASQDIDNYINWYQQKLGKAPNLLIYDASTLETGVPSRFSGSGSGTEYTFTISSLQPEDIATYYCQQYDDLP
CRU          DVQMTQSPSSLSASVGDRVTITCQASQDVTNYVNWYQQKPGKAPKLLIYDASNLETGFPSRFSGSASGTDFTFTIINLQPEDIATYYCQQYDNLP
DEL2 (AFS)   DIQMTQSPSSLSASVGDRVTITCQASQDISNYLNWYQQKPGKAPKLLIHAASSLETGVPSRFSGSGSGTDFSFTISSLQPEDLATYYCQQYDNLP
EPP          DIQVIQSPSSLSASVGDRVTITCQASHDISDHLNWFQQKPGKAPKLLIYDASNLERGVPSRFSGGGSGTDFTFTISSLQPEDIATYYCQQYDDLP
ES305        DIQLTQSPSSLSASVGDRVTITCQASEAIDNYLNWYQQKPGKAPKLLIYNSSNLQTGVPSRFSGSGSGTEFTFTISSLQPEDFATYYCQHYHNLP
GRAV         DILMTQSPSSLSASVGDIVTITCQASQAINKFLNWYQQKPGKAPKLLIYAASNLETGVPLRFSGSGSGTDFTFTISSLQPEDIATYFCQQYDNLP
GRI          DIQMTQSPSSLSASVGDRVTITCQASQDISSYLNWYQQKPGKAPELLIYAGSTLETGVPSRFSGSGSGTDFTFTISSLQPEDVATYYCQQYLNLI
```

```
HIG            DIQMTQSPSSLSASVGDSVTITCQADQDINNSLNWYQQKQGKAPKLLIYDVFHLETGVPSRFSGSGSGTDFTFTISSLQPEDFATYFCQQYNNLP
MALF           DIQMTQSPSSLSASVGDRVTITCQASQDISDYVNWYQQKPGKAPKLLIYDASNLQTGVPSRFSGSGSYTHFTVTIDRLQAEDIATYYCQQNNDFP
MH             DIQMTQSPSYLSASVGDRVNITCQASQDISDHLNWYQQKPGRAPNLLIFGASSLETGVTPRFIGRGYGTHFTFTISSLEPEDVATYYCQQYFNVP
NIG96 (AFS)    DIQMTQSPSSLSASVGDRVTITCQASQDISSYLNWSQQKPGKAPKLLIFEASDLETGVPSRFSGSGSGTDFTFTISSLLPEDIATYYCQQYDNLP
SMA2 (MCN)     DIQMTQSPSSLSASVGDRVTITCQASQDINKYLTWYQQKPGKASKLLIYDASNLETGVPSRFSGSGSGTDFTLTIDNLQPEDIATYYCQQYVNLP
WAT (MCN)      DIQMTQSPSSLSASVGDRVTITCRASQDITNYVNWFQQRPGQAPVLLIYGASILETGVPSRFSGSGSGTDFTFTISSLQPEDIATYYCQQYDTLP
ABU90626       DIQMTQSPSSLSASVGDRVTITCQASQDISNFLIWYQQKPGKAPKLLIYDASNLETGVPSRFSGSGSGTDFTLTISSLQPEDIATYYCQQYNNLP
ABU90662       DIQMTQSPSSLSASVGDRVTITCQASQDIRNYLNWYQQKSGKAPKLLIYDASNLETGVPSRFSGSGSGTHFNLTISTLQPDDTATYYCQQYESLF
ABU90578       DIQMTQSPSSLSASVGDRVTITCQASQDSSYYLNWYQQKPGKAPKLLIYDASNLDTGVPSRFSGSGSGTDFTLTINNLQPEDIATYYCQQYDNVP
ABU90609       DIQMTQSPSSLSASIGDRVTITCQASQDINNYLNWYQQKPGEAPKLLVYDASNLETGVPSRFSGSGSGTDFTFTISSLQPEDIATYFCQQYETLP
ABU90674       DIQMTQSPSSLSASVGDRVSLTCQASQDISTYLIWYQQKSGKAPKLMISGASSLETGVPSRFSGSGSGTHFTFTISSLQPEDIATYYCQQYDYLP
ABU90632       DIQMTQSPSSLSASVGDRVTITCQASHDISKYLGWYQQKPGKAPKLXIYDASNLETGVTSRFSGSGSGTHFTFTINSLQPEDIGTYYCQHYDNLP
ABU90546       DIQMTQSPSSLSASLGDSVTITCQASQGINNFLNWWQQKAGKAPKLVIYDASNLETGVPSRFRGSGTGTHFTFTITNLRPEDIATYYCQQYDTLP
ABU90652       DIQMTQSPSSLSASVGDRVTITCQASQDISNFLNWYQQKPGKAPKLVIHDASNLKTGVPSRFSGTGSGTDFNLTISSLQAEDIATFYCQQFQNLP
ABU90570       DIQMTQSPSSLPVSVGDTVTITCQASQDISIYLNWYQQKPGKAPKFLIYDAANLETGVPLRFSGSGFGANFTFTISSLQTEDIATYYCQQYHNLP
ABU90572       DIQMTQSPSPLSASVGGRVTITCQASQDIYIHLNWYQQKPGEAPKLLIYDVSNLETGVPSRFSGSGSGTDFTFTISSLQPGDIATYYCQQYYNIP
ABU90697       DIQMTQSPSSLSASVGDRVTITCQASQDINKFLIWFQQKPGKAPKLLIYDASNLEIGVPLRFSGSGSGTDFTFTISSLQADDIATYYCQQYDDLP
               ebebebbbeebebbebeebebbbebeeebbeebbbbbbeeeebbebbbbbbeeebeebbebbbbebebbebbbbbebbbeebbbbbbbbbeebb
Match: 2Q20

X93627         DIQMTQSPSSLSASVGDRVTITCRASQSISSYLNWYQQKPGKAPKLLIYAASSLQSGVPSRFSGSGSGTDFTLTISSLQPEDFATYYCQQSYSTP
AM107          DIQMTQSPSSLSASVGDRVTITCRASQTSSLDLNWYQQKPGKAPKLLIYADSSLQTGVPSRFSGSGSGTDFTLTISGLQPEDFATYFCQQYDTGP
AM113          DIQMTQSPSSLSASVGNRVTITCRASQSSVDYVAWYQQKPGKAPKLLIFDASSLQSGVPSRFSGTGSGTDFTLTISSLQPEDFATYYCQQFDNLP
AND            DIQMTQSPSSLSASVGDRVTITCRASHSIGGSLNWYHQKPGKAPSLLIFGASSLRSGVPSRFSGSGSGTDFTLTITSLQPEDFAAYFCQQSYSSP
CHEB (AFS)     DIQMTQSPSSLSASVGDRVTFTCRASQTIATFLNWYQQKPGKAPKLLIYGASSLQSGVPSRFSGSGSGTDFTLTISSLQPEDFATYYCQQSYSIP
COZ            DIQMTQSPSSLAASVGDRVTITCRARQSISNYLNWYQHKPGKAPNVLIYAASNLQSGVPSGFSGSGSGTEFTLTITSLQPEDFATYYCQQSYSAP
CRO            DIQMTQSPSSLSASVGDRVTITCRASQSFNNFLNWYQQKPGKAPKLLIYDASTLESGVPSRFSGSGSGTDFTLTISSLQPEDFATYYCQQTYTGI
CRO2 (MCN)     DIQLTQSPSSLSASVGDRVSVTCRASQSISSYLHWYQQKPGKAPELLIYGASSLQNGVPSRFSGIGSGTDFTLTISNLQPEDFATYYCQQTYSSP
DRU (MCN)      DIQMTQSPSSLSAFVGDRVTITCRASQSISTYLNWYQQKPGKAPELLIYAVSTLQSGVPSRFSGSGSGTDFTLTISSLQPEDFASYYCQQSYSMP
MEV            DVQMTQSPSSLSASVGDRVIITCRASQSSVDYLNWYQQKPGKAPKLLIFDTSNLQSGVPSRFSGGRSGTDFTLTISSLQPDDFATYYCQQSYTNP
MUM            DIQLTQSPSSLSASVGDRVTITCRASQSINSYLNWYQQKPGKAPNLLIYDASSLQSGVPSNFSGSGSGTDFTLTIRSLQREDFATYYCQQSYSIP
TRE (AFS)      DIQMTQSPSSLSASVGDRVTITCRASQSIAGYLNWYQQRPGKAPELLIYAASTLRSGVPSRFSGSGSGADFTLTISSLQPEDSATYYCQQSYSYP
TRO2 (AFS)     DIQMTQSPSSLSASVGDRVTITCRASQSNISYLNWYQQKPGKAPKLLMYAASSLQSGVPARFVGSGSGTDFTLTISSLQPEDFATYYCQQTYSAP
ABU90684       DIQMTQSPSSLSASVGDRVTITCRASQSISSYLNWYQQKQGKAPNLLIYATSSLQSGVSPRFSGSGSGTNFTLTISSLLPDDFATYYCQQSYSTP
ABU90556       DIRLTQSPSSLSASVGDRVTISCRASQSVSTYLNWYQQKPGKAPKLLIYAASNLQSGVPSRFSGSGSETDFTLTISSLQAEDFAIYYCQQGYSSP
ABU90543       DIQMTQSPSSLSASVGDRVTITCRASQSISDYLNWYQQKPGKAPNLLIYAASSVQSGVPSRFSGSGSGTDFTLTISSLQAEDFATYYCQQSSNAP
ABU90642       DIQMTQSPSSLSASVGDRVTITCRASQRISKSLNWYLQKQGQAPQLLISSASSLQSGVPSRFSGGGSGTDFSLTISNLQAEDFATYYCQQSYTVP
ABU90636       DIQMSQSPPSVSASVGDRVTITCRASQNIISYINWYQQRPGKAPKLLIYAASSMQSGVPSRFSGSGSGTEFTLTINTLQPEDFGTYYCQQSYSNP
ABU90633       DIQMTQSPPSLSASVGDRVTLTCRASQGIGNSLNWYQQKPGQAPKVLIYAASSLQSGVPSRFSGSGSGTDFTLIISSLQREDSGTYYCQQSYSTP
ABU90598       DIQMTQSPSSLSASVGDGVTITCRASQTISTSVNWYQQNPGKAPKLLIYGVSNLQSGVPSRFSGSGSGTEFTLTISSLQPEDFATYYCQQSYSNP
               ebebebebeebebeebeebebbbebeeebbbbbbbbbbeeeebeebbbbbbebebeebbebbebebebbebbbbbebbbeebbbbbbbbbebeee
Match: 2BX5

X93632         DIVMTQSPLSLPVTPGEPASISCRSSQSLLHSNGYNYLDWYLQKPGQSPQLLIYLGSNRASGVPDRFSGSGSGTDFTLKISRVEAEDVGVYYCMQALQTP
```

```
K2HUTW         DIVMTQSPLSLPVTPGEPASISCRSSQSLLHSDGFDYLNWYLQKPGQSPZLLIYALSNRASGVPDRFSGSGSGTDFTLKISRVEAEDVGVYYCMZALQAP
DRE            DIVMTQSPLYLPVIPGEPASISCRSSQSLLHSNGNNYLDWYLQKPGQSPKLVIYLGSNRASGVPDRFSGSGSGTDFTLKINKVEAEDVGVYYCIQALQTD
INC            DIVLTQSPLSLPVTPGEPASISCKSSQSLMHSSGDNYLDWYLQKPGQSPQIVIYLGSNRASGVPDTFSGSGSGTDFTLLISSVGAEDVGVYYCMQAEQTP
NIC            DIVMTQSPLSLPVTPGEPASISCRSSQSLLHTNGYNYLDWYLQKPGQSPQLLLYLGSNRVSGVPDRFSGSGSGTNFTL---RVEAEDVGTYYCMQGLQIP
TEW            DIVMTQSPLSLPVTPGEPASISCRSSQSLLHSDGFDYLNWYLQKPGQSPQLLIYLGSNRASGVPDRFSGSGSGTDFTLKISRVEAEDVGVYYCMQALQAP
               ebebebbbeebebbebbebbbbbebeeebebeebbbbbbbbbbbeeebbebbbbbbebebeebbebbbbebebbebbbebbebeeebbbbbbbbbbbeb
Match: 1CBV
```

```
X93638         EIVMTQSPATLSVSPGERATLSCRASQSVSSNLAWYQQKPGQAPRLLIYGASTRATGIPARFSGSGSGTEFTLTISSLQSEDFAVYYCQQYNNWP
AAD09376       EILMTQSPANLSVSAGEGATLSCRASQSISINLAWYQQRPGQAPRLLIFDASTRAPRSPDRFSGSGSGTEFTLTISSLKSEDSATYYCQQYNNWP
AL700          EIVMTQSPATLSVSPGGRATLSCWASQSVTSNLAWYQQHPGQAPRLLIYATSTRATGTPARFSGSGSGTQFTLTISSLQSEDFAVFYCQQYNNWP
LIGN (LCDD)    EIVMTQSPGTLSVSPGDRASLSCRASQDVGSSLAWYQQKPGQAPRLLIYDASTRASGLPARFSGSGSGTEFTLTISSLQSEDFAVYYCQQHHTWP
LONG           EVVMTQSPVTLSVSPGERATLSCRASQSVISKLAWYQQKPGQAPRLLVYDASTRATGIPGRVSGSGSGTEFTLTSSGLQSEDSAVYYCQQYYRWS
REV            EIILTQSPATLSVSPGERATLSCRASQSVSTNLAWYQQKPGQAPRLLIYGASIRATGISARFSGSGSGTEFTLNITSLQSGDLALYFCQQYGDWP
SCI            EIVMTQSPGTLSVSPGERATLSCRASQSVSYNLAWYQQKPGQAPRLLIYSTSIRATGIPARFSGSGSETEFTLTISILQSEDFAVYYCQHYEDWP
SO124          EIVMTQSPATLSVSPGERVTLSCRASQSVRVNLAWYQQKPGQAPNFLIYGSTTRATGIPARFSGSGSGTEFNFTISSLQSEDFAVYYCQQYNNWP
               ebebbbeeeebebbebeebebebebeeebeebbbbbbbbeeebbbbbbbbbebebeebbebbbbebebbebbbbbebbebbbbbbbbbbbbbeb
Match: 1RZ8
```

```
X93640 (B3)    DIVMTQSPDSLAVSLGERATINCKSSQSVLYSSNNKNYLAWYQQKPGQPPKLLIYWASTRESGVPDRFSGSGSGTDFTLTISSLQAEDVAVYYCQQYYSTP
1EK3_A         DIVMTQSPDSLAVSPGERATINCKSSQNLLDSSFDTNTLAWYQQKPGQPPKLLIYWASSRESGVPDRFSGSGSGTDFTLTISSLQAEDVAVYYCQQYYSTP
1LVE = LEN     DIVMTQSPDSLAVSLGERATINCKSSQSVLYSSNSKNYLAWYQQKPGQPPKLLIYWASTRESGVPDRFSGSGSGTDFTLTISSLQAEDVAVYYCQQYYSTP
AAD09378       ELVMTQSPDSLAVSLGERATINCKSSRSVLSDSNSRNLLAWFQQKPGQPPKLLIYWASTRVSGVPDRFSGSGSGTDFTLTISSLQAEDVAVYYCQQYYSTP
P83593         DIVMTQSPDSLVVSLGERATINCRSSQSVLYSSNNKNYLAWYQQKPGQAPKLLFSWASTRESGVPDRFSGSGSGTDFTLTIPGLQAEDVAVYYCQQYYRIP
BLU            DIVMTQSPDSLAVSLGERATINCKSSLSVFFSPNNKNYLAWYQQKPGQPPKLLIYWASTRESGVPDRFSGSGSGTNFTLTISRLQAEDVAVYYCQQYYTTL
BURN           DIVMTQSPDSLAVSLGERATINCKSSQSVLYNSNNKNFLAWYQQKPGQPPKLLIYWASTRGSGVPDRFSGSGSGTDFTLTITRLQAEDVAVYYCQQYYSTP
CAB            DIVMTQSPDSLSVSLGERATINCKSSQSVLLSFKNDNYLAWYQQKPGQSPK---YWASTRESGVPDRFSGSGSGTNFTLTISSLQAEDVAVYYCQQYYTTP
FRA            DIVMTQSPDSLAVSLGERATINCKSSQSLLYSTRNKICLAWYQQKPGQTPQLLISWASSRESGVPDRFSGSGSGTDFSLTISSLQAEDVATYYCQQYSTAP
KAL            DIVMTQSPDSLAVSLGERATINCKSSQSVLYSSNNKNYLAWYQQKPGQPPKLLIYWASTRQSGVPDRFSGSGSGTDFTLTISGLQAEDVAVYYCQQYYGTP
REC            DIVMTQSPDSLAVSLGERATINCKSSQNVLDASFDTNTLAWYQQKPGQPPKLLIYWASSRESGVPDRFSGSGSGTDFTLTISSLQAEDVAVYYCQQYYSTP
SMA            DIVMTQSPDSLAVSLGERATINCKSSQSVLYSSNNRNYLAWYQQKLGQPPKLLIYWASTRESGVPDRFSGSGSGTDFTLTISSLQAEDVAVYYCHQYYSHP
ABU90663       DIQMSQSPPSVSASVGDRVTITCRASQNIISYINWYQQRPGKAPKLLIYAASSMQSGVPSRFSGSGSGTEFTLTINTLQPEDFGTYYCQQSYSNPRTFGPG
ABU90562       AIVMTQSPDSLTVSLGERATINCKSSHNLFFSSNNKNYLAWYQQIPGQPPKLLLYWASTRESGVPDRFNGSGSGTDFTLSISSLQAEDVAVYSCQQYYSAP
ABU90713       DIVMTQSPDSLAVSLGERATINCKSSQTVLYSSNNKNYLAWYQQKPGQPPKLLIYWASTRESGVPDRFSGSGSGTDFTLTISSLQAEDVAVYSCQQYYSIP
ABU90648       DIVMAQSPDSLAVSLGERATINCKSSQNVFYSSNNKNYVAWYQQKPGQPPRLLIYRASTRESGVPDRFSGGGSGTDFTLTISSLQAEDVAVYFCQQYYTTP
ABU90646       DIVVTQSPDSLAVSPGERATINCKSSQSVLHSSYNANYLAWYQQKQGQPPRLLIYWASTRESGVPDRFSGSGSGTDFTLTISSLQAEDVAVYYCQQYYTTP
ABU90637       DIVMTQSPDSLTVSQGERATINCRSSQNLLYSSNNRNYLAWYQQRPGQPPKLLIDWASTRESGVPDRFSGSGSGTHFTLTISSLQAGDVAVYYCQQYYSIP
Match: 1LVE
```

Lambda sequences

```
M30446 (humlv114)    QSVLTQPPS-VSAAPGQKVTISCSGSSSNIGNNYVSWYQQLPGTAPKLLIYENNKRPSGIPDRFSGSKSGTSATLGITGLQTGDEADYYCGTWDSSLSA
A29700               QLVLTQPPS-VSAAPGQKVTISCSGSSSNIGNNYVSWYQQLPGTARKLLIYEDNKRPSGIPDRFSGSKDATVATLGITGLQTGDEADYYCGTWDSSLSA
AAC97078             QSVLTQPPS-VSAAPGQKVTISCSGSYSNIGKNYVSWYQHIPGTAPRLLIFENNKRPSGIPDRMSASKSGPSATLDIAGLQTGDEADYYCATWENSLTA
```

```
AIR                  QSVLTQPPS-VSAAPGQKVTISCSGSSSNIANNYVSWYQQLPGAAPKLLIYENVKRPSGIPDRFSGSKSGTSATLGITGLQTGDEAEYYCGTWDSSLSG
ALX                  --VLTQPPS-VSAAPGQKVTISCSGSSSNIGNNSVSWYQQFPGAAPKLLIYDNHKRPSRIPDQFSGSKSGTSATLGITGLQTGDEADYYCGTWDNSLNT
EPS                  --VLTQPPS-LSAAPGQRVSISCSGSSSNIGKNSVDWYQQLPGTAPKLLIFNNNKR------RFSGSKSGTSATLGITGLQTGDEAIYYCGTYDNRRSV
EZI                  -SVLTQPPS-VSAAPGQTVTISCSGSSSNIGNNYVSWYQQFPGTAPKLLIYDDNKRPSGIPDRFSGSKSATSATLGITGLQTGDEADYYCGAWDSRFSA
ZIM                  -SLLTQPPS-VSAAPGQKVTISCSGSSSNIGNNYVSWYQQLPGTAPRLLIYENDKRPSGIPDRFSGSKSDASVTLGITGLQTGDEADYYCGTWDSSLSA
                     ebebebbee bebbeeeebebebbbbebbbbebebbbbbbbbebebebbbbebeebbeebbebbbbbbeebbbbbbbebbeebbbbbbbbbbbbeebbb
Match: 1RZF

Z22188 (HUMLV1L1)    QSVLTQPPS-ASGTPGQRVTISCSGSSSNIGSNTVNWYQQLPGTAPKLLIYSNNQRPSGVPDRFSGSKSGTSASLAISGLQSEDEADYYCAAWDDSLNG
Cox                   SVLTQPPS-ASGTSGQRVTISCSGSSSNIGSNQVNWYRHLPGTAPKLVIYSDSQRPSGVPDRISASKSGTSASLAISGLQSEDESDYYCASWDDSLDG
Mcph                  SVLTQPPS-ASGTPGQRVTIACSGSSSNIGSNAVTWYQQLPGTAPQLLIDTNNQRPSGVPDRFSGSKSGTSASLAISGLQSEDEADYYCAATDDSLNG
Dia                   SVLTQPPS-ASGTPGQRVTISCSGSSSNIGSNVVTWYQQLPGTAPKLLIYTNNQRPSGVPGRFSGSKSGTSASLAVSGLQSEDEADYYCATWDDSVNG
Emm                   SVLTQPPS-ASGTPGQRVTISCSGSSSNIRSNTVNWYQHVPGAAPKLLIYINNQRPSGVPDRFSGSKSGTSASLAISGLQSDDEADYYCATWDDSLNG
Loc (MCN)             SVLTQPPS-ASGTPGQRVTISCSGSSSNIGENSVTWYQHLSGTAPKLLIYEDNSRASGVSDRFSASKSGTSASLAISGLQPEDETDYYCAAWDDSLDV
Nig51                 SVLTQPPS-ASGVPGQSVIISCSGSSSNIGRNTVNWYQQVPGAAPKLLVYSNNQWPSGVPDRFSGSKSGTSASLAISGLHSEDEADYFCATWDDSLDG
Nig77                 SVLTQPPS-ASGTPGQRVTISCSGSTSNIGSNTVTWYQHLPGTAPKLLIYSNDQRPSGVPHRFSGSKSGASASLAISGLQSEDETDYYCATWDDSLNG
Pfu                   SVLTQPPS-ASGTPGQRVTLSCSGGSSNLGVNTVNWYQQLPGADPKVLIYRNDQRPSGVPDRFSGSKSGTSASLAISGLQSEDEADYHCAAWDDSLNG
A42193               QSVLTQPPS-ASGTPGQRVTISCSGSSSNIGSNVVTWYQQLPGTAPKLLIYTNNQRPSGVPGRFSGSKSGTSASLAVSGLQSEDEADYYCATWDDSLNG
AAC97079             QSVLTQPPS-ASGTPGQRVTISCSGSNSNIGINIVNWYQQVPGRAPKLLIYSNNQRPSGVADRFSGSKSGTSASLAISGLQSEDEADYYCAAWDDSLHG
AAC97080             QSVLTQPPS-ASGTPGQRVTISCSGSSSNIGSNTVTWYQHLPGAAPKLLIYSNDQRPSGVPDRFSGSKSGTSASLAISGLQSVDEAHYYCSAWDDRLNG
AAC97081             QSVLTQPPS-ASGTPGQRVTISCSGSRTNIASNTVNWYQHLPGMAPKLLIYTNDQRPSGVPDRISGSKSGTSASLGISGLQSEDEADYYCAAWDDRLNG
AAC97082             QSVLTQPPS-ASGTPGQRVSISCSGSTSNIGSNTVNWYLHLPGTAPKLLIYSNNQRPSGVPDRFSGSKSGTSASLAISGLQSEDEADYYCAAWDDSLDG
AAD29289             QSVLTQPPS-ASGTPGQRVTISCSGSNSNIGSNSVNWYQHLPGTAPKLLIYSQSQRPSGVPDRFSGSRSGTSASLAISGLQSEDEADYYCAAWDDSLNG
AAD29290             QSVLTQPPS-ASGTPGQRVIISCSGSSSNIGRNTVNWYQHLPGTAPKLVIYSNDQRPSGVPDRFSGSKSVTSASLAISGLQSGDEAHSYCAAWDDSLRG
ABU90645             QSVVTQPPS-ASGTTRQRVTISCSGGSSNVGSNTVNWYQHLPGTAPKLLIYSGNQRPSGVPDRFSASKSGTSXSLAISGLQSDDEGDYYCAVWDDSLNG
ABU90651             QSVLTQPPS-ASGTPGQRVTISCSGSSSNIGSNTVNWYQHLPGTAPKFLIFSNNQRPSGVPDRFSGSKSGTSASLAISGLQSEDEADYYCAAWDDSLNG
ABU90658             QSMLTQPPA-ASGTPGQRVSISCSGSSSNIGSNTVNWYQQLPGTGPKLLIFSSNQRPSGVPDRFSGSKSGTSASLAISGLQSEDEADYYCAAWDDSLNG
ABU90681             QSVLTQPPS-ASGNPGQRVTISCSGTISNIGSNTVNWYQHLPGTGPKLLIFSNNQRPSGAPDRFSGSRSGTSASLAISGLRSEDEADYFCAAWDDTLNG
ABU90708             QSVLTQPPS-ASGTPGQRVFVSCSGSSSNIGSNTVNWYQHLPGTAPKLLIYITDQRPSGVPDRFSGSKSGTSASLAITGLQSEDEADYYCAVWDDSLNG
ABU90545             QSVLTQPPS-ASGTPGQRVTISCSGSSSNLGSNTVNWYQHLPGSAPKLLIYSNNQRPSGVPDRFSASKSGTSASLAISGLQSEDESNYYCAAWDARLKG
ABU90657             QSVLTQPPS-ASGTPGQRVTISCSGSISNIGSNTVNWYQQLPGTAPKLLMYSNDQRPSGVPERFSGSKSGTSVSLAISGLQSEDEADYYCAAWDDSLNG
ABU90559             QSLLTQPPS-ASGTPGQRVTISCSGRRSNIGSNTVTWYQQLPGTAPKLLIYTNDLRPSGVPDRFSGSKSGTSGSLAISGLQSADEGDYYCAAWDDSLNG
ABU90664             QSVLTQAPS-ASGTPGQTVTISCSGSRSNIGSNTVNWYQQVPGTAPTFLIYSNSQRPSGVPDRFSGSKSGTSASLAISGLQSDDEADYYCAAWDDSLNG
ABU90635             QSVLTQPPS-ASGTPGQRVTISCSGSTSNIGTNTVNWYQHVPGAAPQLLIYTNDQRPSGVPDRISGSKSGTSASLAISGLQSEDEADYHCAAWDDSLHG
ABU90693             QSVLTQPPS-ASGTPGQRVTISCSGRSSNIGSNTVHWYQHVPGAAPKLLIYSNDERPSGVPDRFSGSKSGTSASLAISRLQSEDEAHYYCATWDDSPNA
ABU90715             QSVLTQPPS-ASGAPGQTITISCSGSSSNIGINTVCWYQQYPGTAPKLLIYTNDHRPSGVPDRFSGSKSGTSASLAISGLQSEDEADYYCAAWDDILDG
ABU90617             QSVLTQPPS-ASGTPGQVVTMSCSGSSSNIGGNTVAWYQHLPGAAPRLLVYSNSERPSGVPDRFSGSKSDTSASLAISGLQSEDEADYYCAAWDDTLNG
ABU90619             QSVLTQPPS-ASGTPGQSITISCSGSSSNIGSNSVTWYQHLPGTAPKLLIYSVNQRPSGVPARFSGSKSGTSASLAIIGLQSEDEADYYCGAWDGSLSG
ABU90601             QSVLTQPPS-ASATPGQRVTISCSGSSSNIGNNYVYWYQHLPGTAPKLLIYANNQRPSGVPDRFSGSKSGTSTSLAISGLRSEDEADYHCAAWDDSLSG
ABU90692             QSVLTQPPS-ASGTPGQRVTISCSGSSSNIGSNFVYWYQQVPGTAPKFLIYSNNQRPSGVPDRFSGSKSGTSASLAISGLRSEDEADYYCAAWDDSLSV
ABU90589             QSVLTQPPS-ASGTPGQGVTISCSGSSSNIGSNYVYWYQYLPGMAPKLLIYLNNQRPSGVPDRFSGSKSGTSASLAISGLQSGDESDYYCATWDDSLNE
ABU90597             QSVLTQPPS-ASGTPGQRVTISCSGSSSNIGINYVYWYQQFPGTAPRLLIYTNNQRPSGVPDRFSGSKSGISASLAISGLRSDDEANYYCATWDDSLGG
ABU90571             QSVLTQPPS-VSETPGQTVTISCSGSSSNIGSVYVYWYQHLPGTAPKLLIQRNSQRPSGITDRFSGSKSGTSASLTISGLRSEDEADYYCAVWHDSLSG
                      eebebbeb bbbeeeeebebbbebeebbbbebbbbbbbbeeebbbebbbeebebebeebbebbbbebeebbbbbbbbbbeeebebbbbbbbebeeeeb
```

```
Match: 4BJL (second match)

Z22191        QSVLTQPPS-VSAAPGQKVTISCSGSSSN-IGNNYVSWYQQLPGTAPKLLIYDNNKRPSGIPDRFSGSKSGTSATLGITGLQTGDEADYYCGTWDSSLSA
AAD09370      QSVLTQPPS-VSAAPGQKVTISCSGNRYN-IGNNYVSWYQQFPGTAPKLLIYDDNERPSGIPDRFSGSKSGTSATLGITGLQTGDEADYFCGTWDSGLTA
AAD29291      QSVLTQPPS-VSAAPGHKVTISCSGSSSN-IGKNYVSWYQQIPGTSPKLLIYDNNKRPSGIPARFSGSKSGTSATLVITGLQSGDEADYYCGTWDSSLSD
AAD29292      QSVLTQPPS-VSAAPGQNVSISCSGSSSN-IGNNHVSWYQQFPGTAPKLLTYDNNERPSGIPDRFSGSKSGTSATLVITGLQTGDEADYFCGTWDYTLS-
AAD29293      QSVLTQPPS-VSAAPGQKVTISCSGSSSD-IGNNYVSWYQQLPGAAPKLLIYDTSKRPSGVPDRFSGSNSGTSATLGITGLQTGDEADYYCGTWDGSLSA
L1HUEP        QSVLTQPPS-LSAAPGQRVSISCSGSSSN-IGKNYVDWYQQLPGTAPKLLIFNNNKRPSGIPDRFSGSKSGTSATLGITGLQTGDEAIYYCGTWDNRRSV
              ebebebbee bebbeeeebebebbbbebbbbebebbbbbbbbebebebbbbebeebbeebbebbbbbbeebbbbbbbebbeebbbbbbbbbbbeebbb
Match: 1RZF

Z22197        QSALTQPAS-VSGSPGQSITISCTGTSSDVGGYNYVSWYQQHPGKAPKLMIYEVSNRPSGVSNRFSGSKSGNTASLTISGLQAEDEADYYCSSYTSSSTL
AAC97083      LSELTQPAS-VSGSPGQSITISCTGTSSDVAIYNYVSWYQQYPTKAPKLTIYEVNNRPSGVSNRFSGSKSGNTASLTISGLQAEDEADYYCSSYTITGTQ
AAC97085       SELTQPAS-VSGSPGQSITISCTGTSSDVGGYNYVTWYQQHPGKAPNVLIYEVTYRASGVSNRFSGSKSGNTASLTISGLQAEDDADYYCSSYTINSTR
AAD29294       SELTQPAS-VSGSPGQSITISCTGTSNDVGGYTYVSWYQQHPGKAPKVIIYDVSNRPSGVSNRFSGSKSGNTASLTISGLQAEDEADFYCSSYTSSSTV
AAD33349      QSALTQPAS-VSGSPGQSITISCTGTSSDIGNYNYVSWYRQHPGKAPKLIIYDVTNRPSGVSNRFSGSKSGNTASLTISGLQAEDEADYYCNSYTTSDTL
L2HUNG        QSALTQPAS-VSGSPGQSITISCTGTTSDVGGYDFVSWYQQHPGKAPKLLIYDVNSRPSGISNRFSGSKSGNTASLTISGLQAEDEADYYCSSFTTTNSR
JOHN              TQPAS-VSASPGQSITISCTGTSTDVGVYNFVSWYQQHPGKAPKVMIYDVSNRPSGVSNRFSGSKSGNTASLTISGLQAEDEADYYCSSYTSSST
MC             SALTQPAS-VSGSPGQSITISCTGTSSDVGGYNYVSWYQQHPGKAPQLMIYEVSNRPSGVSNRFSGSKSGNTSSLTISGLQAEDEADYYCSSYTIGTR
NIG84          SALTQPAS-VSGSPGQSITISCTGTTSDVGGYDFVSWYQQHPGKAPKLLIYDVNSRPSGISNRFSGSKSGNTASLTISGLQAEDEADYYCSSFTSTNSR
NOV            SALTQPAT-VSGSPGQSITISCTGTSSDVGDYNYVSWYQQHPGKAPKLMIYDVSDRPSGVSYRFSGSKSGNTASLTISGLQAEDEADYYCSSYTSDST
ABU90665      QSALTQPAS-VSGSPGQSITISCTGTSSDVGGYNYVSWYQQHPGKAPKLMIYDVSYRPSGVSDRFSGSKSGNTASLTISGLQAEDEADYYCNSYTSSSTP
ABU90590      QSALTQPAS-VSGSPGQSITISCTGTSSDVGGYNFVSWYQQHPGKAPKLMIFDVSNRPSGVSHRFSGSKSDNTASLTISGLQAEDEADYYCSSYTSSSTW
ABU90568      QSALTQPAS-VSGSPGQSITISCTGTSSDVGGYNYVSWYQHHPGKAPKFMIYDVSQRPSGVSNRFSGSKSGNTASLTISGLQAEDEADYYCSSYTGSSTY
ABU90606      QSALTQPAS-VSGSPGQSITISCTGTSSDVGGYNYVSWYQHHPDKAPKLMIYEVSNRPSGVSNRFSGSKSGNTASLTISGLQAEDEADYYCSSYTSTSDV
ABU90704      QSALTQPAS-VSESPGQSITIYCAGTSSDVGGYDYVSWYQQHPGKAPKLIIYDVSDRPSGVSNRFSGSKSGNTASLTISGLQAEDEADYYCSSYTSTDTL
ABU90729      QSALTQPAS-VSESPGQSITISCAGTSSDVGNYDYVSWYQQHPGKVPKVIIYDVNNRPSGVSNRFSGSKSGNTASLTISGLQAEDEADYYCSSYSSSSAF
ABU90667      QSALTQPAS-VSGSPGQSITISCTGTSSDVGNSDSVSWYQQHPDKVPKLMIYDVSNRPSRVSDRFSGSKSGNTASLTISGLQTEDEADYYCSSYAASGYV
ABU90661      QSALTQPAS-VSGSPGQSITISCTGTSSDVGNYNYVSWYQQHPGKAPKLMIYDVGNRPSGVSNRFSGSKSGNTASLTISGLQAEDDGDYYCLSHTNNSTL
ABU90577      QSALTQPAS-VSGSPGQSITISCTGTSSDNNDYKYVSWYEQHPGKAPKLMIYEVSSRPSGVSNRFSGSKSGNTASLTISGLRAEDEADFYCSSHINKTLV
ABU90702      QSALTQPAS-VSGSPGQSITISCTGSSSDVDTYKYVSWYQHHPGKAPKLMIYDVSNRPSGISNRFSGSKSGNTASLTISGLRAEDETDYYCSSYTRNNTL
ABU90622      QSALTQPAS-VSGSPGQSITISCTGTSHDVGGYDDVSWYQQHPGKAPKLMIYDVRNRPSGVSNRFSGSKSGNTASLTISGLQAEDEADYYCTSHTSRNTL
ABU90605      HSALTQPAS-VSGSPGQSISISCTGTSSDVGGYNYVSWYQHYPGKAPKLIIYEVTNRPSGVSNRFSGSKSGNTASLTISGLQAEDEADYFCDSYTSDSTW
ABU90701      QSALTQPAS-VSGSPGQSITISCTGSISDVGGYNFVSWYQQHPGKAPKLMIYDINNRPSGVSNRFSGSKSGNTASLTISGLRAEDEADYYCNSYTVSSSS
ABU90655      QSALTQPAS-VSGSPGQSITISCTGTSSDVGGYKYVSWYQQHPGKAPKVMIYEVSNRPSGVSNRFSGSKSGNTASLTISGLQAEDEADYYCNSYTNSSNY
ABU90618      QSALTQPAS-VSGSLGQSIIISCTGSASDVGGYDFVSWYQQYPGKAPKVMIYDVSSRPSGISNRFSGSKSGNTASLTISGLQAGDEADYYCSSYTSSSTY
ABU90593      QSALTQPAS-VSGSPGQSITISCTGSSNDVGYNYVSWYQQHPGKGPKVMIYDVSSRPSGVSNRFSGSKSGNTASLTISGLQAEDEADYYCTSYTRTNTYV
ABU90639      QSVLTQPAS-VSGSPGQSITISCTGTSNDVGGYNYVSWYQQLPGKAPKLVIYDVDKRLSGVSSRFSGSKSGNTASLTISGLQADDEATYHCISYISSSAL
ABU90575      QSALTQHAS-VSGSPGQSITISCTGTNSDIGLYNYVSWYQHHPGKAPKLLIYDVIKRPSGVSTRFSGSKSGNTASLTISGLQAADEATYSCNSYTANTTF
              eeebebbeb bbbbeeeebebbbebeeebbbbbebbbbbbbeeebbbebbbbebebebeebbebbbbebeebbbbbbbbbbbeebbbbbbbbbbbeeebb
Match: 2OLD

Z22208        SYELTQPPS-VSVSPGQTASITCSGDKLGDKYACWYQQKPGQSPVLVIYQDSKRPSGIPERFSGSNSGNTATLTISGTQAMDEADYYCQAWDSSTA
AAC97087      SYELTQPPS-VSVSPGQTASITCSGDRLGDKFACWYQQKPGQSPVLVIYEDTKRPSGIPERFSGSNSGNTATLTISGTQAMDEGDYYCQSWDSSTA
```

```
AAD09368     SYELTQPPS-VSVSPGQTASIPCSGDKLGDKYVCWYQQKPGQSPVVVIYEDNKRPSGIPERFSGSNSGNTATLTISGTQAMDEAYYSCQSWDTTTA
AAD09369     SYELTQPPS-LSVSPGQTASITCSGDKLEDNYVSWYQQKPGQSPVVVINQDYKRPSGIPERFAGSNSGNTATLTISGTQAVDEADYYCQVWASSSD
AAD09374     SYELTQPPS-VSVSPGQTATITCSGDKLGDRYVCWYQHKPGQSPALVVYQDSQRPSGIPERFSGSNSGNTATLTISGTQGMDEADYYCQAWDSSAA
AAD29296     QSVLTQPPS-VSVSPGQTASITCSGDKLGSEYVCWYQQKAGQSPVLVIYEDTKRPSGIPDRFSGSNSGNAATLTISDTQAMDAADYYCQTWDSSTA
AAD29297     QSVLTQPPS-VSVSPGQTGSITCSGEKLGDKYACWYQQKPGQSPVVVIYQDNMRPSGIPERFSGSNFGNTATLTISGTQAMDEADYYCQAWDSGTA
CLE (MCN)    SYEVTQPPS-LSVSPGQTARITCSGEKLGDAYVCWYQQRPGQSPVVVIYQDNRRPSGIPERFSGSSSGNTATLTISGTETLDEADYYCQVWDSNAS
DOD          SYELVQPPS-VSVSPGQTARITCSGDKLGDRFASWYQQKPHQSPMLIIYQDNKRPSGIPERFSGSNSGNTATLTISGTLAVDEADYHCEAWDGNTA
DOY          SFDLTQPPS-VSVSPGQTASITCSGEKLGEEYAAWYQQKPGQSPVLVIYQDTKRPSGIPERFSGSNSGNTATLTISGTQALDEADYYCQAWDSSSV
MOL          SYELTQPPS-VSVSPGQTATISCSGDKLGEXXYDWYQQSPGQSPLLVIYEGDKRPSGXXXRFSGSNSGNTATLTISGTESMDEADYYCQAWNSSSV
NIG68 (AFS)  SYDLTQAPS-LSVSPGQTAYITCSGDNLGNEFVSWYQQRPGQSPALVIYDTSKRPSGIPERFSGSKSGNTATLTISGTESMDEADYYCQAWDQIRD
NIG95        SYDLTQPPS-VSVSPGQTATISCSGENLGDNFACWYQQKPGQSPVLVIYQDNKRPSGIPERFSGSNSGNTVTLTITGNTTMDEADYYCQVWDVGAV
WHIT         -YELAQPPS-VSVSPGQTASITCSGEKLGDKYTWWYQQKTGQSPVLVIYQDTKRPSGIPERFAGSNSGNTATLTISGTQAMDEADYYCQTWDNTRV
ABU90588     SYELTQPPS-VSVSPGQTASITCSGDKLGDKYACWYQQKAGQSPVLIIYQDSKRPSGIPERFSGSNSGNTATLTISGTQAMDEADYYCQAWDSSTG
ABU90608     SYELTQPPS-VSVSPGQTASITCSGDKLGDKYACWYQQKPGQSPVLVIYQDSKRPSGIPERFSGSNSGNTATLTISRTQALDEADYYCQAWDSSTC
ABU90682     SYELSQPPS-VSVSPGQTASITCSGDKLGDIYACWYQQKSGQSPVLVIYQDTRRPSGIPERFSGSTSGNTVTLTISGTQAIDEADYYCQAWDSNTV
ABU90710     SYELIQPPS-VSVSPGQTASITCSGDKLGDKYACWYQQKPGQSPVLVIYQDTRRPSGIPERISGSNSGNTATLTISGTQATDEADYYCQAWDSSTV
ABU90723     SYELTQPPS-VSVSPGQTASITCSGDKLGDKFACWYQQKPGQSPVLVIYQDRERPSGIPERFSGSNSGNTATLTISGTQTNDEADYFCQAWDSDTS
ABU90691     SYDLTQPPS-VSVSPGQTASITCSGDKLGDEYACWYQLKPGQSPVLVIYEDTKRPSGIPERFSGSNSGNTATLTISGTQAMDEADYFCQAWDSGTV
ABU90615     SYELTQPPS-VSVSPGQTASITCSGDKLGDKYACWYQQKPGQSPVLVIYQASKRPSGIPERFSGSNSGNTATLTISGTQTMDEADYYCQAWDSSTV
ABU90558     SYELTQPPS-VSVSPGQTASITCSGDKLGNEYACWYQQKPGHSPVLVIYQDSKRPTGIPERFSGSNSGNTATLTISGTQAVDEADYYCQTWDFNTA
ABU90656     SYELTQPPS-VSVSPGQTAIITCSGDKLGSKYAWWYQQKPGLSPVLVIYQDNKRPSGIPERFSGSNSGNTATLTVSGTQDMDEADYYCQTWDSSNV
ABU90630     SYDLTQPPS-VSVSPGQTASITCSGAKLGDEYASWYQQKPGQSPILVIYQDGKRPSGIPERFSGSNSGNTATLTISGAQAMDEADYFCQAWDSSSV
ABU90563     SYDLTQPSS-VSVSPGQTASITCSGNKLGNKYVWWYQQKPGQSPVLVIYQDAKRPSGIPERFSASNSGNTATLTIGGTQAMDEADYYCQTWDISTV
ABU90685     SFELTQPPS-VSVSPGQTASITCSGDKLGDEYVCWYQQKPGQSPLVVIYQDDKRPSGIPERFSGSSSGNTATLTITGTQSMDEADYYCQAWDNSAA
ABU90669     SYELTQPPS-VSVAPGQTASITCSGDKLDNKYISWFQQKPGQSPLLVMYQDNKRPSGIPERLSGSSSGNTATLTISGTQAMDEAAYYCQAWDSNTG
ABU90561     SYELTQPPS-VSVSPGQTASITCSGHKLGDTYTCWYQQKPGQSPLLVIYQDNKRPSGIPERFSGSNSGNTATLSISGTQALDEADYFCQAWDSSTA
ABU90679     SPEVTQPPS-VSVSPGQTASITCFGDKLGDEYVSWYQQKPGQSPVLVIYKDSRRPSGIPERFSGSNSGNTATLTISGTQTLDEADYYCQTWDDATA
ABU90677     SSELSQPPS-VSVSPGQTASITCSGDVLGGKYACWYHQKAGQSPMLLIYQDSKRPSGIPDRFSGSNSGNTATLTISGTQALDEADYYCQAWDSSIA
ABU90566     PYELTQPPS-VSVSPGQTASITCSGDTLGNNFASWYQQKPGQSPVLVIYQDTKRPSTIPGRFSGANSGNTATLTISGTQTIDEGDYYCQTWDRSTL
ABU90732     SYDLTQPPS-VSVSPGQTASITCSGDNLGDQYACWYQQKPGQSPVLIIYQDNKRPSGIPERFSGSNSGDTATLTISGTQSTDEADYFCQTWANNSA
ABU90624     SYELTQPPS-ISVSPGQTASITCSGDKLGAKYTSWYQQKPGQSPVLVIYQDYRRPSGIPERFSGSNSGNTATLTISGTQAVDDGDYYCQAWDSSTG
ABU90607     SYELTQPPS-VSVSPGQTASITCSGDNLGDKYASWFQQKPGQSPRLVMYQDSKRPSGIPERFSGSNSGNTATLTISGTQTMDEADYYCQAWDSTTA
ABU90595     SYDLTQPPS-VSVSPGQTATISCSGNNLGNKYVSWYQQKPGQSPVLVIFQDDKRPSGIPERFSGSNSGNTATLTISGTQAADEADYYCQAWDSSTP
ABU90553     QSVLTQPPS-VSVSPGQTASIPCSGDKLGVKYVSWYQQRPGQSPVLVMSQDTERPSGVSERFSGSNSGNTATLIISGTQAMDEADYYCQVWDSSTV
             eeebebbbb bbbbebeebebbbbbeebbebebbbbbbeeeebbbbbbbebeebbbebeebbbbeeeeebbbbbebbeeebbbbbbbbbbbbebb
Match: 1LIL
```

```
Z73673       NFMLTQPHS-VSESPGKTVTISCTRSSGSIASNYVQWYQQRPGSSPTTVIYEDNQRPSGVPDRFSGSIDSSSNSASLTISGLKTEDEADYYCQSYDSSN
1CD0_A       NFMLNQPHS-VSESPGKTVTISCTRSSGNIDSNYVQWYQQRPGSAPITVIYEDNQRPSGVPDRFAGSIDRSSNSASLTISGLKTEDEADYYCQSYDARN
2CD0_A       NFLLTQPHS-VSESPGKTVTISCTRSSGSIASNYVHWYQQRPGSSPTTVIFEDDHRPSGVPDRFSGSVDTSSNSASLTISGLKTEDEADYYCQSYDHNN
AAC97090     NFMLTQPHS-VSESPGKTVTISCTRNSGSIASNYVQWYQQRPGSSPNIVIYEDNVRPSGVPDRFSGSIDSSSNSASLTISELKTEDEADYYCQSYDNNN
AAC97091     NFMLTQPHS-VSESPGKTVTISCTRSSGSIATSYVQWYQQRPGSSPTTIIFEDNLRPSGVPDRFSGSIDSSSNSASLTISGLRTEDEADYYCQSYDSGK
AAC97092     NFMLTQPHS-VSESPGKTVTISCTRSGGSIAGDYVQWYQQRPGSAPTSVIYEDDQRPSGVPDRFSGSIDSSSNSASLTISGLKTEDEADYYCQSFDNNN
AAC97093     DFMLTQPHS-VSESPGKTVTISCTGSSGSIASNYVQWYQQRPGSAPTTVIYEDDQRPSGVPDRFSGSIDTSSNSASLTISRLKTEDEADYYCQSYDSNN
```

```
AAC97094      NFMLTQPHS-VSESPGKTVTISCTRSSGSIASNYVQWNQQRPGSAPSTVIYEDSQRPSGVPDRFSGSIDTSSNSASLTISGLKTEDEADYYCQSYDGSN
AAC97095      DFMLTQPHS-VSESPGKTVTISCTRSSGSIASNYVQWYQQRSGNSPSAVIYEDNQRPSGVPDRFSGSIDSSSNSASLTISGLMTEDKADYYCQSYSGSN
AAC97096      DFMLTQPHS-VSESPGKSVTISCTGSSGSVAVNDVQWYQQRPGSAPTTVIYDDDQRSSGVANRFSVSIDSSSNSASLTISGLKTEDEADYYCQSFDSNN
AAD28579      NFMLTQPHS-VSESPGKTVTISCTRSSGSIVSNYVQWYQQRPGSAPTTVISEDDQRPSGVPDRFSGSIDSSSNSASLTISGLRTEDEADYYCQSYDSSS
AAD29298      DFMLTQPHS-VSESPGKTVTISCTGSSGSIANNYVQWYQQRPGSAPTTMIYEDNQRPSGVPDRFSGSIDSSSNSASLTISGLKTEDEADYYCQSFDNSK
AAD29299      DFMLTQPHS-VSESPGKTNTISCTRSTGSIASNYVQWYQQRPGSAPFTLIYEDNQRPSGVPDRFSGSIDSSSNSASLSISGLKTEDEADYYCQSFADFT
AAD29300      DFMLTQPHS-VSESPGKTVTISCTRSSGSIANNYVQWYQQRPGSSPTNVIHEDNQRPSGVPDRFSGSIDSSSNSASLTISGLKAEDEADYYCQSYDSTK
AAD29301      DFMLTQPHS-VSESPGKTVTISCTRSSGNIASNYVQWYQQRPASSPTTVIYEDNQRPSGVPDRFSGSIDSSSNSASLTISGLKTEDEADYYCQSYDTNI
AAK58585      NFMLTQPHS-VSGSPGKTITISCTGSSGRIASNSVQWYQQRPGSAPNIVMYENNQRPSGVPDRFSGSIDSSSNSASLTISGLMTEDEADYYCQSFDDST
AAK58586      NFMLTQPHS-VSESPGKTITISCTRSSGSIASNYVQWYQQRPGSAPTTVIYEDNQRPSGVPDRFSGSIDSSSNSASLTISGLKTEDEADYYCQSYDSNN
AAK58587      NFMLTQPHS-VSESPGKTVTISCTGSSGSIATNYVQWYQLRPGSAPTTVIYEDNQRPSGVPDRFSGSIDSSSNSASLTISGLKTEDEADYYCQSYDSSI
L6HUAR        DFMLTQPHS-VSESPGKTVTFSCTGSGSIASDSFVQWYQQRPGSAPTTVIYDDNQRPSGVPDRFSGSIDDSANSASLTISGLKTEDEADYYCQSYNSNH
L6HULT        NFMLTQPLS-VSGSPEKTVTISCTGSSGSIGSNYVQWYQQRPGSAPTNVIYENNQRPSEVPDRFSGSIDSSSNSASLTISGLKTEDEADYYCQSYDNNN
CROS          DFMLTQPHS-VQESPGKTVTISCTRSSGSIASNYVQWYQQRPGRAPTTMIYEDNQRPSGVPDRFSASIDSSSNSASLTISGLKTEDEADYYCQSYDNSN
GARN          NFILTQPHS-VSESPGKTVTISCTVSGGNIGKNYMQWYQQRPNSAPTTVIFENNQRPSGVPLRFSGSIDASSNSASLTIVDLKTEDEAEYFCQTFADTN
GIO           NFMLTQPHS-VSESPGKTVTISCTGSSGSIASNFVQWYQQRPGSAPATVIYEDNQRPSGIPDRFSGSIDSSSNSASLTISGLKTEDEADYYCQSYDSTH
JONE          NFMLTQPHS-VSESPGKTVTISCTRSSGSLASTYVQWFQQRPGSAPTTVIYEDNQRPSGVPDRFSGSIDSSSNSASLTINGLKTEDEADYYCQSYDSET
K11           NFMLTQPHS-VSDSPGKTVIISCTRSSGNIATNYVQWFQQRPGSAPTTVIYDDNQRPSGVPDRFSGSIDRSSNSASLTISGLKPEDEADYYCQSYDATS
MAL2          KFMLTQPHS-VSASPGKTVTISCTRSSGSIDTNYVQWYQQRPGSAPTNIIYEDNQRPSGVPDRFSGSIDSSSNSASLTISGLKTEDEADYYCHSYDSST
QUI           NFMLTQPHS-VSESPGKTVTISCTRSSGNIASNYVQWYQQRPGSSPTIVIFEDDQRPSGVPDRFSGSIDSSSNSAELTISDLKTEDESDYYCQSYDSNY
RUD           NFMLTQPHS-VSESPGKTVTISCTRSSGSIASNYVQWYQQRPGSAPTTVIFENAQRPSGVPDRFSGSIDSSSNSASLTISGLKTADEADYYCQSYDNSN
SUT           DFMLTQPHS-VSESPGKTVIISCTRSSDSTAGYYVQWYQQRPGRAPTTVIFEDTQRPSGVPDRFSGSIDRSSNSASLTISGLQTEDEADYYCQSYDRDH
THO           NFMLTQPHS-VSESPGKTVTISCTRSSGSIASYYVQWYQLRPGSAPTTVIYEDNQRPSGVPDRFSGSIDRSSNSASLTVSELKTEDEADYYCQSYDSNN
WILD          NFLLTQPHS-VSESPGKTVTISCTRGNGSIANNYVHWYQQRPGSSPTTVIFEDDHRPSGVPDRFSGSVDTSSNSASLTISGLKTEDEADYYCQSYDHNN
WIND          NFMLTQPHS-VSESPGKTVTISCTRSSGSIASYFVQWYQQRPGSSPTTNIYEDTERPSGVPDRFSGSIDSSSNSASLTISGLRTEDEADYYCQSFDSSN
JTO (MCN)     NFMLNQPHS-VSESPGKTVTISCTRSSGNIDSNYVQWYQQRPGSAPITVIYEDNQRPSGVPDRFAGSIDRSSNSASLTISGLKTEDEADYYCQSYDARN
ABU90621      NFMLTQPHS-VSESPGKTVTISCTRITGSVASNFVQWFQQRPGSSPTTVIYEDDQRPSGVPDRFSGSIDSSSNSASLTISGLKTEDEADYYCQSYDGST
ABU90596      NFMLTQPHS-VSESPGKTVTISCTRSSGSIASNYVQWFQQRPGSSPSTVIYEDDQRPSGVPDRFSGSVDSSSNSASLTISGLKTEDEADYYCQSSDSSD
ABU90698      NFMLTQPHS-VSESPGKTVTISCTRSSGSIASNYVQWYQQRPGRSPTVVIYEDNERPSGVPDRFSGSIDSSSNSASLTISGLKTEDEADYYCQSYDSNI
ABU90706      NFMLTQPHS-VSESPGKTVTISCTRSSGSIASNYVQWYQQRPGSSPTIVIYEDNQRPYGVPDRVSGSIDSSSNSASLTISGLKTDDEADYYCQSYDGGH
ABU90711      NFMLTQPHS-VSESPGKTVTISCTRNSGSIASNYVQWYQQRPGSPPTTVIYEGNQRLSGVPARFSGSIDSSSNSASLTISGLRTEDEADYYCQSYDISS
ABU90695      NFMLTQPHS-VSESPGKTVTISCTRSSGSIASYYVQWSQQRPGSAPTTVIYKDNQRPSGVPDRFSGSIDSSSNSASLTISGLKTEDEADYYCQSYDTNK
ABU90714      NFMLTQPHS-VSESPGKTVTISCTRSGGNIANNYVQWYQQRPGSAPTTVIYEDNLRPSGVPDRFSGSIDSSSNSASLTISGLKTEDEADYYCQSYDTSN
ABU90689      NFILTQPHS-VSESLGKTVIISCTGSSGSIASNYVQWYQQRPGSAPTTVIYEDNQRPSGVPDRFSGSIDRSSNSASLTISGLKTEDEADYYCQSYAGSS
ABU90576      QFMLTQPHS-VSESPGKTVTISCTRSSGNIDSNYVQWYQHRPGSSPTTVIYEDDQRPSGVPDRFSGSIDRSSNSASLSISGLKTEDEADYYCQSYDARS
ABU90565      DFMLTQPHS-VSESPGKTVTISCTRSSGSIASNYVQWYQQRPGSAPTTVIYEDDQRPSGVPARFSGSIDRSSNSASLTISGLQTEDEADYYCQSYERVF
ABU90612      NFMLTQPHS-VSGSPGKTVTISCTGSSGSIASNYVQWYQQRPGSAPTAVIYEDDRRPSGVPDRFAGSIDTSSNSASLTISGLKTEDEADYYCQSFDSFN
ABU90587      NFVLTQPHS-VSESPGKTVTISCIRSSGIISNNYVQWYQQRPGSSPTIVIYEDSQRPSGVPDRFSGSIDSSSNSASLTISGLKTEDEADYYCQSYGSFN
ABU90551      NFMLTQPHS-VSESPGKTVTISCTRSSGSIASTYVQWYQQRPGSAPTNVIFEDNERPSGVPDRFSGSIDSSSNSAYLTISGLKTEDEADYYCQSYGTNN
ABU90567      NFMLTQPHS-VSESPGKTVTISCTRSSGSIASNYVQWYQQRPGSAPTTVIYENNQRPSGVPDRFSGSVDSSSNSASLTISGLKTEDETDYYCQSYDRFN
              ebbbbbbbe bebbeeeebbbbbbbbbbebebbebbbbbbeeebbbbbbbbbbeeebeebbebbbbebbeeebbbbbbbbbbeeebebbbbbbbbbbeeb
Match: 3BDX
```

Non-amyloidogenic sequences

```
J00248 (L1)  DIQMTQSPSSLSASVGDRVTITCRASQGISNYLAWFQQKPGKAPKSLIYAASSLQSGVPSRFSGSGSGTDFTLTISSLQPEDFATYYCQQYNSYP
AAY33383.1   DIQMTQSPSSLSASVGDRVTITCRASQGISNYLAWYQQKPGNAPKSLIYAASSLESGVPSKFSGSGSGTDFTLTISSLQPEDFATYYCQQANSFP
AAW69259.1   DIQLTQSPSSLSASIGDRVTITCRASQGIGNSLAWFQQKPGKAPKSLIYAASSLQSGVPSKFSGSGSGTDFTLTISSLQPEDFATYYCQQYKTYP
AAD56280.1   DIQMTQSPSSLSASVGDRVTITCRASQGISNYLAWYQQKPGKVPKSLIYAASTLQSGVPSRFSGSGSGTDFTLTISSLQPEDVATYYCQKYNSAP
AAY33422.1   DIQLTQSPSSLSASVGDRVTITCRASQGISSHLAWYQQKPGKAPKLLIYAASSLQSGVPSRFSGSGSGTEFTLTISSLQPEDFATYYCQQFNSYP
AAY33480.1   DIQMTQSPSSLSASVGDRVTITCRASQGISSWLAWYQQKPGKAPKSLIYAASSLQSGVPSKFSGSGSGTEFTLTISSLQPDDFATYYCQQLNSYP
AAB26430.2   DIQMTQSPSSVSASVGDRVTITCRASQGISSWLAWYQQKPGKAPKLLIYAASSLQSGVPSRFSGSGSGTDFTLTISSLQPEDFATYYCQQANSFP
AAF05690.1   ELQMTQSPPSLSASVGDRVTITCRASQGISTYLVWYQQKPEKAPKSLIYAASNLQSGVPSRFSGSGSGTDFTLTISSLQPEDFATYYCQQYNSYP
AAY33461.1   DIQMTQSPSSLSASVGDRVTITCRASQGISSALAWYQQKPGKAPKLLIYDASSLESGVPSRFSGSGSGTDFTLTISSLQPEDFATYYCQQFNSYP
AAY33423.1   DIQMTQSPSSLSASVGDRVTITCRASQGIRNDLGWYQQKPGKAPKLLIYAASSLQSGVPSRFSGSGSGTDFTLTISSLQPEDFATYYCQQLNSYP
AAY33484.1   DIQLTQSPSSVSASVGDRVTITCRASQGISSWLAWYQQKPGKAPKLLIYAASSLQSGVPSRFSGSGSGTDFTLTISSLQPEDFATYYCQQANSFP
AAW69123.1   DIQMTQSPSSLSASVGDRVTITCRASQDITNYIAWFQQKPGKAPKSLIYAASTLQSGVPSKFSGSGSGTEFTLTISSLQPEDSATYYCQQYDGYP
AAY33464.1   DIQLTQSPSSLSASVGDRVTITCRASQGISSYLAWYQQKPGKGPKLLIYAASTLQSGVPSRFSGSGSGTDFSLTISSLQPEDLATYYCQQYDSYP
AAW67411.1   DIQVTQSPSFLSASVGDRVTITCRASQGISSYLAWYQQKPGKAPKLLIYAASTLQSGVPSRFSGSGSGTEFTLTISSLQPEDFATYYCQQLNSYP
AAB34102.1   DIQVTQSPSSLSASVGDRVTITCRASHDIGSYLAWYQQKPEKAPESLIYAASSLQSGVPSRFSGSGSGTDFTLTISSLQPEDFATYYCQQYNSYP
```

Lambda sequences

```
M30446       QSVLTQPPS-VSAAPGQKVTISCSGSSSNIGNNYVSWYQQLPGTAPKLLIYENNKRPSGIPDRFSGSKSGTSATLGITGLQTGDEADYYCGTWDSSLSA
CAA84394     QSVLTQPPS-VSAAPGQRVTISCSGSSSNIGSDYVSWYQQLPGTAPKLLIYDNNKRPSGIPDRFSGSKSGTSATLAITGLQTGDEADYYCGTWDSSLSF
AAY33470     QSVVTQPPS-VSAAPGQKVTISCSGSSSNIGDKSVSWYQQLPGTAPKLLIYENDKRPSGIPDRFSGSKSGTSATLGITGLQTGDEADYYCGTWDSSLSA
AAR88382     QSVLTQPPS-VSAAPGQKVTISCSGSSSNIGNNDVSWYQQFPGTVPKLVIYENNQRPSGIPDRFSGSKSGTSATLGITGLQTGDEADYYCGTWDSSLSA
CAD30279     QSVLTQPAS-VSGSPGQSITISCSGSSSNIGNNYVSWYRQLPGTAPKLLIYDNNKRPSGIPDRFSGSKSGTSATLGITGLQTGDEADYYCGTWDSSLSA
CAA54621     QSVLTQPPS-VSAAPGQKVTISCSGNSSNIGNNYVSWYQHLPGTAPKLLIYDNNKRPSSIPDRFSGSKSGTSATLGITGLQTGDEADYYCATWTSILRV
AAA85072     QSVLTQPPS-MSAAPGQKVTISCSGSSSNIGNNYVSWYQQFPGTAPKLLIHDNNKRPSGIPDRISGSKSGTSASLAISGLQSDDEADYYCAAWDDSLNG*
AAB27503     QSVLTQPPS-ASGTPGQRVTISCSGSSSNIGRNVVNWYQQLPGTAPKLLIYSNNQRPSGVPDRFSGSKSGTSASLAISGLQSEDEADYYCATWDDSLTG*
AAP31882.1   QSVLTQPPS-ASGTPGQRVTISCSGSSSNIGSNYVYWYQQLPGTAPKLLIYRNNQRPSGVPDRFSGSKSGTSASLAISGLRSEDEADYYCAAWDDSLSG*
ABA55013     QSVLTQPPS-ASGTPGQRVTISCSGSSSNIGSNYVYWYQQLPGTAPKLLIYRNNQRPSGVPDRFSGSKSGTSASLAISGLRSEDEADYYCAAWDDSLSA
```

```
X72813       DIQMTQSPSTLSASVGDRVTITCRASQSISSWLAWYQQKPGKAPKLLIYKASSLESGVPSRFSGSGSGTEFTLTISSLQPDDFATYYCQQYNSYS
BAB18253.1   DIQMTQSPSTLSASVGDRVTITCRASQSISSWLAWYQQKPGKAPKLLIYKASGLESGVPSRFSGSGSGTEFTLTISSLQPDDFATYYCQQYNSYW
1DFB         DIQMTQSPSTLSASVGDRVTITCRASQSISRWLAWYQQKPGKVPKLLIYKASSLESGVPSRFSGSGSGTEFTLTISSLQPDDFATYYCQQYNSYS
AAT76800.1   QIQMTQSPSTLSASVGDRVTITCRASQSISSWLAWYQQKPGKAPKLLIYKASSLESGVPSRFSGSGSGTEFTLTISSLQPDDFATYYCQQYNSYP
AAW69061.1   EIVMTQSPSTLSASVGDRVTITCRASQSISTWLAWYQQKPGKAPKLLIYKASSLESGVPSRFSGSGSGTEFTLTISSLQPDDFATYYCQQYNSYF*
AAY33355.1   DIQLTQSPSSLSASVGDRVTITCRASQSISSWLAWYQQKPGKAPKLLIYDASSLESGVPSRFSGSGSGTEFTLTISSLQPDDFATYYCQQFNNYP
AAL59374.1   DIQMTQSPSTLSASVGDRVTITCRASQTITNWLAWYQQKPGRAPKLLIYKASNLESGVPSRFSGSGSGTEFTLTISSLQPDDFATYYCQHYNNYP
BAB18255.1   DIELTQSPSTLSASVGDRVTITCRASQSLNNWLAWYQQKPGKAPKLLIYKASSLESGVPSRFSGSASGTEFTLTISSLQPDDFATYYCQQYNSPW
AAW69258.1   DIQLTQSPSTLSASVGDRVTITCRASQSISSWLAWYQQKAGKAPTLLIYEASTLQSGVPSRFSGSGSGTEFTLTISSLQPDDFATYYCQQYNPYS
AAW69129.1   DIQLTQSPSTLSASVGDRVTITCRASQSVSSWLAWYQQKPGKAPKVLISRASDLESGVPSRFSGSGSGTEFTLTISSLQPDDFATYYCQQYKTYS
AAW68991.1   DIQMTQSPSTLSASVGDRVTITCRASQSIGSWLAWYQQKPGKAPKLLIYKASTLQSETPSRFRGSGSGTEFTLTISSLQPDDFATYYCQQFNSFS
```

```
AAW68897.1    DIQLTQSPSTLSASVGDRVTITCRASQSIGSWLAWYQQKPGKAPKLLIYKASTLQSETPSRFRGSGSGTEFTLTISSLQPDDFATYYCQQFNSFS
AAO91637.1     IVLTQSPSTLSASVGDRVTITCRASQSISSWLAWYQQKPGKAPKLLIYKASNLQSGVPSRFSGSGSGTEFTLTISSLQPDDFASYYCQQYSNYP
AAB27975.1|   DIQMTQSPSTLSASVGDRVTITCRASLSISSWLAWYQQKPGIAPKLLIYQASSLQTGVPSRFSGSGSGTEFTLTISSLQPEDFATYYCRQHNSYP
1RZG          DIQMTQSPSTLSASVGDRVTITCRASQSISNWLAWYQQKPGRAPKLLMYKASSLKSGVPSRFSGSGSGTEFTLTISSLQSDDFATYYCQQHDSSP
CAI99816.1    -HRMSQSPSTLSASVGDRVTITCRASQSLNGWLAWYQQKPGKAPKLLIYKASSLESGVPSRFSGSGSGTEFTLTISSLQPDDFATYYCQHYXGYP
AAX57560.1    ----TQSPSTLAASVGDRVTITCRASQFIDTWLAWYQQKPGKAPKLLIYKASTLQSGVPSRFSGSGSGTEFTLTISSLQPDDFATYYCQRYNSHS
AAT96426.1    DIVMTQSPSTLSASVGDRVTITCRASQSIDIWLAWYQQKPGKTPKLLIYKASTLESGVPSRFSGSGSGTEFTLTISSLKPDDFASYYCQHYNRDS*
AAW69083.1    EIVMTQSPSSLSAFVGDRVTITCRANQTFSSWLAWYQQKPGRAPKLLIYKASTLEGGVPSRFSGSGSGTESTLTISSLQPDDFATYYCQQYNSFS
```

```
X93620        DIQMTQSPSSLSASVGDRVTITCQASQDISNYLNWYQQKPGKAPKLLIYDASNLETGVPSRFSGSGSGTDFTFTISSLQPEDIATYYCQQYDNLP
PH0864        DIQMTQSPSSLSASVGDRVTITCQTSQDISKYLNWYQQKPGKAPKLLIYDASNLERGVPSRFSGGGSGTDFTFTISSLQPEDIATYYCQQYDTLP
P01608        DIQMTQSPSSLSASVGDRVTITCQASQDISIFLNWYQQKPGKAPKLLIYDASKLEAGVPSRFSGTGSGTDFTFTISSLQPEDIATYYCQQFDNLP
AAD47066.1    DVVMTQSPSSLSASVGDRVTITCQSSLDISHYLNWYQQKPGKAPKLLIYDASNLETGVPSRFSGSGSGTHFTFTISSLQPEDFATYYCQQYDNLP
AAY33403.1    DIQLTQSPSSLSASVGDRVTITCQASQDISNYLNWYQQKPGKAPKLLIYDASNLETGVPSRFSGSGSGTDFTFTISSLQPEDFATYYCQQSYSSP
AAO91641.1    EIVLTQSPSSLSASVGDRVTITCQASQDISNYLNWYQQKPGKAPKLLIYDASNLETGVPSRFSGSGSGTDFTFTISSLQPEDFGTYYCQQYNTYP
AAW69058.1    DIQLTQSPSSLSASVGDRVTITCQASRDITNYLNWYQLKPGKAPKLLIYDASNLATGVPPRFSGSRSGTDFSFTISSLQPEDVATYYCQQYDTLP
AAW69119.1    DIQMTQSPSSLSASVGDRVTITCQASQDITNYLNWYQLKPGKAPKLLIYDASNLATGVPPRFSGSRSGTDFSFTISSLQPEDVATYYCQQYDTLP
AAW68974.1    DIQMTQSPSSLSASVGDRVTITCQASQDITNYLNWYQQKPGKAPKLLIYDASNLQPGVPSRFSGSRSGTDFTFTISSLRPEDIATYYCQQYDGPV
AAW69032.1    DIQLTQSPSSLSASVGHRVTITCQASQDITNYLNWYQLKPGKAPKLLIYDASNLATGVPPRFSGSRSGTHFTFTISSLQPEDVATYYCQQYDTLP
AAW69009.1    DVVMTQSPSSLSASVGDRVSITCQASQDITNYLNWYQQKPGKAPKLLIYDASNLQPGVPSRFSGSRSGTDFTFTISSLRPEDIATYYCQQYDGPV
AAD19529.1    ----TQSPSSLSASVGDRVTITCQASQDISNFLTWYQKKPGKAPKVLIYDASNLETGVPSRFSGSGSGTDFTFTIASLQPDDFATYYCQQYNDHP
AAQ22022.1    --------SSLSASVGDRVTITCQASQDIRKYLNWYQQKPGKAPKLLIYDASNLEIGVPSRFSGSGSGTDFTFTISSLQPEDIATYYCQQYDNLY
AAS79808.1    ----TQSPSSLSASVGDRVTITCQASQDISNYLNWCQQKPGKAPKLLIYDGSYLETGVPSRFSGTGSGTDFTFTISSLQPEDIATYYCQQYDNLP
```

```
X93627        DIQMTQSPSSLSASVGDRVTITCRASQSISSYLNWYQQKPGKAPKLLIYAASSLQSGVPSRFSGSGSGTDFTLTISSLQPEDFATYYCQQSYSTP
1DEE          DIQMTQSPSSLSASVGDRVTITCRTSQSISSYLNWYQQKPGKAPKLLIYAASSLQSGVPSRFSGSGSGTDFTLTISSLQPEDFATYYCQQSYSAP
AAV66330.1    DIQMTQSPSSLSASVGDRVTITCRASQSISSYLNWYQQKPGKAPKLLIYSASSLQSGVPSRFSGSGSGTDFTLTISSLQPEDFATYYCQQSSSNP
AAV73916.1    DIQMTQSPSSLSASVGDRVTITCRASQSISSYLNWYQQKPGKAPKLLIYSASYLQSGVPSRFSGSGSGTDFTLTISSLQPEDFATYYCQQNYNSP
AAY33396.1    DIQMTQSPSTLSASVGDRVTITCRASQGISSWLAWYQQKPGKAPKLLIYAASSLQSGVPSRFSGSGSGTDFTLTISSLQPEDFATYYCQQSYSTP
AAR91610.1    DIQMTQSPSSLSASVGDRVTITCRASQSISSYLNWYQQKPGKAPKLLIYNASSLQSGVPSRFSGSGSGTDFTLTISSLQPEDFATYYCQQDTNTP
AAY33454.1    DIQMTQSPSSLSASVGDRVTITCRASQTISSYLNWYQQKPGKAPELLIYAASRLQSGVPSRFRGSGSGIDFTLTISSLQPEDFATYYCQQSYSTP
AAV73914.1    DIQMTQSPSSLSASVGDRVTITCRASQSISSYLNWYQQKPGKAPKLLIYSASALQSGVPSRFSGSGSGTDFTLTISSLQPEDFATYYCQQDSSAP
1RZI          DIQMTQSPS-LSASVGDRVTITCRASQSISSYLNWYQQKPGKVPKLLIYAASSLQSGVPSRFSGSGSGTDFTLTISSLQPEDFATYYCQQSYSTS
AAW69005.1    DIQMTQSPSSLSASLGDRVTITCRASQHISNYLNWYQQKPGKAPKLLIYAASSLQSGVPSRFTGSGSGADYTLTISSLQPEDFATYYCQQSYSTS
AAC13460.1    AE-LTQSPSSLSASVGDRVTITCRASQNISSYLNWYQQKPGKAPKLLIYAASSLQSGVLSRFSGSGSGTDFTLTISSLQPEDFATYYCQQSYSTP
AAB30087.1    DIQMTQSPSSLSASIGDRVTITCRASQSIIKYLSWYQQKPGRAPKLLIYTASSLQGGVPSRFSGSGSGTDFTLTISSLQPEDFATYYCQQNYNTP
AAB26310.1    -E-LTQSPSSLSASVGDRVTITCRASQSISNYLNWYQQKPGKAPKLLIYAASSLQRGVPSRFSGSGSGTDFTLSISSLQPEDFATYYCQQSYSIP
AAY33481.1    DIQLTQSPSSVSASVGDRVTITCRASQGISRHLNWYQQKPGKAPKLLIYDASNLETGVPSRFSGSGSGTDFTLTISSLQPEDFATYYCQQSYSTP*
```

```
X93632        DIVMTQSPLSLPVTPGEPASISCRSSQSLLHSNGYNYLDWYLQKPGQSPQLLIYLGSNRASGVPDRFSGSGSGTDFTLKISRVEAEDVGVYYCMQALQTP
AAW63081.1    ----TQSPLSLPVTPGEPASISCRSSQSLLHANGYNYLDWYLQKPGQSPQVLIYLGSNRASGVPDRFSGSGSGTDFTLKISRVEAEDVGVYYCMQALQTP
AAK61518.1    ELVMTQSPLSLPVTPGEPASISCRSSQSLQQSNGHNYLNWYLQKPGQSPQFLIHLGSNRASGVPDRFSGSGSGTDFTLKISRVEAEDVGVYYCMQALQTP
AAB25910.2    DIVMTQTPLSLSVTPGQPASISCKSSQSLLHSDGKTYLYWYLQKPGQSPQLLIYEVFNRFSGVPDRFSGSGSGTDFTLKISRVEAEDVGVYYCMQSIQLP
ABC67123.1    DIVMTQSPLSLPVTPGEPASISCRSSQSLLHRNGYNYLDWYLQKPGQSPQLLIYLGSNRAFGVPDRFSGSGSGTDFTLKISRVEAEDVGVYYCMQALQTR
```

```
ABC66952.1   DIVMTQSPLSLPVTPGEPASISCRSNQSLLYSNGYNYLDWYLQKPGQSPQLLIYSGSNRASGVPDRFSGSGSGTDFTLKISRVEAEDVGVYYCMQALQSP
CAA84373.1   DIVMTQSPLSLPVTPGEPASISCRSSQSLLHSNGNNYLDRYLQKPGQSPQLLIYLGSNRASGVPDRFSGSGSGTDFTLKISRVEAEDVGVYYCMQALQTP
CAA73230.1   DIVMTQSPLSLPVTPGEPASISCRSSQSLLHSDGYNSLDWFLQRPGQSPQLLIYLGSNRASGVPDRFSGSGSGTDFTLKISRVEAEDVGVYYCMQVLQTP
ABA71394.1   IFVMTQSPLSLPVTPGEPASISCRSGQSLLHSNGYNYLDWYLQKPGQSPQLLIYLGSNRASGVPDRFSGSGSGTDFTLKISRVEAEDVGVYYCMQSLRTR

X93640       DIVMTQSPDSLAVSLGERATINCKSSQSVLYSSNNKNYLAWYQQKPGQPPKLLIYWASTRESGVPDRFSGSGSGTDFTLTISSLQAEDVAVYYCQQYYSTP
AAW67406     EIVMTQSPDSLAVSLGERATINCKSSQSVLFSSNNKNYLAWYQHKPGQPPKLLLYWASTRESGVPDRFSGSGSGTDFTLTITSLQAEDVAVYYCQQYFYTP
CAA85510     DIVMTQSPDSLALSLGERATINCKSSQSVLYSSNNKNYLAWYQQKPGQPPKLLIYWASSRESGVPDRFSGSGSGTDFTLTISSLQAEDVALYYCHQFFTSP
AAW69250     DVVMTQSPDSLAVSLGERATINCKSSQSVLYDSNNKNYLSWYQQKPGQPPKLLLKWASTRESGVPDRFSGGGSGTDFTLTISSLQAEDVAVYFCQQYYRSP
AAS60097     ---LTQSPDSLAVSLGERATINCKSSQSILDSSNNRNYLTWYQQKPGQPPKLLIYWASTRESGVPDRFSGSGSSTDFTLTISSLQAEDVAVYYCQQYFSTP
AAS47840     DIVMTQSPDSLAVSLGERATINCKSSQSVLYTTKNKNHLAWYQQKPGQPPKLLIYWASTRESGVPDRFSGSGSGTDFSLTITNLQAEDVALYYCHQYYLFP
AAQ21859     -------PDSLAVSLGERATINCKSSQTVLYSSNNKNYLAWYQQKPGQPPRLLIYWATTRESGVPDRFSGSGSGTDFTLTISSLQAEDVALYYCQQYYSSP
AAZ32310     DILVTQSPGSLAVSLGERATINCKSSQSILYSSNNKNYLAWYQQKAGQPPKLLISWASTRESGVPDRFSVSGSGADFTLTISSLQAEDVAVYYCQQYYNTP
CAI99841     FIQLTQSPDSLAVSLGERATINCKSSQSILYTSNNKNYLAWYQQKPGQPPKLLIYLASTRESGVPDRFSGSGSGTDFTLTINSLQAEDVAVYYCQQYYIYP
AAS47840     DIVMTQSPDSLAVSLGERATINCKSSQSVLYTTKNKNHLAWYQQKPGQPPKLLIYWASTRESGVPDRFSGSGSGTDFSLTITNLQAEDVALYYCHQYYLFP
CAJ31438     DIVMTQSPDSLAVSLGERATITCKSSQSLLYRFKNKNFLAWYQQKPGQPPKLLIYWASTRESGVPDRFSGSGSGTDFALTISSLQAEDVAIYYCQQYYSSV
AAT96492     DIVMTQSPDSLAVSLGERATISCRSSQSVLYGPNNKNYLAWYQQRLGQPPKLLIYWGSTRESGVPDRFSGSGSGTDFTLTISSLQAEDVAVYYCQQYYSNL
AAZ09078     DIVMTQSPDSLAVSLGERATINCRSSQSLLYSSNNHNKLAWYQQKPGQPPKLLIYRASTRESGVPDRFRGSGSGTDFTLTISSLQAEDVAVYYCQQYYSPP
ABB55182     DIVMTQSPDSLAVSLGERATINCKSNQSLLYSSNNNNYLTWYQQKPGQPPKLLFYWAYTRGSGVPDRFSGGGSGTDFTLTISSLQAEDVAVYYCQQYYTSP

Z22188       QSVLTQPPS-ASGTPGQRVTISCSGSSSNIGSNTVNWYQQLPGTAPKLLIYSNNQRPSGVPDRFSGSKSGTSASLAISGLQSEDEADYYCAAWDDSLNG
AAP31882     QSVLTQPPS-ASGTPGQRVTISCSGSSSNIGSNYVYWYQQLPGTAPKLLIYRNNQRPSGVPDRFSGSKSGTSASLAISGLRSEDEADYYCAAWDDSLSG
AAN16433     QSVLTQPPS-ASGTPGQRVTISCSGSSSNIGSNYVYWYQQLPGTAPKLLIYRNNQRPSGVPDRFSGSKSGTSASLAISGLRSEDEADYYCAAWDDSLVF
AAN02492     QSVLTQPPS-ASGTPGQRVTISCSGSSSNIGSNYVYWYQQLPGTAPKLLIYRNNQRPSGVPDRFSGSKSGTSASLAISGLRSEDEADYYCAAWDDSLAS
AAS19429     SYELTQPPS-ASGTPGQRVTISCSGSSSSIGSETVNWYQQLPGTAPKLLIYSNNQRPSGVPDRFSGSKSGTSASLAISGLQSEDEADYYCTAWDDTLNG
AAB68783     QSVLTQPPS-ASGTPGQRVTISCSGSSSNIGSNYVYWYQQLPGTAPKLLIYRNNQRPSGVPDRFSGSKSGTSASLAISGLRSEDEADYYCAAWDDRLSG
AAK84192     QSVLTQPPS-ASGTPGQRVTISCSGSSSNIGSNYVYWYQQLPRTAPKLLIYRNNQRPSGVPDRFSGSKSGTSASLAISGLRSEDEADYYCAAWDDSLGF
AAY33378     QSVLTQPPS-ASGTPGQRVTMSCSGGSSNIGRDNVYWYQQLPGTAPKLLIYNDIQRPSGVPDRFSGSKSGTSASLAISGLQSEDEADYYCAAWDDSLSG
AAW67400     QPVLTQPPS-ASGTPGQRVTISCSGSSSNIGSNYVYWHQQLPGTAPKLLIYRNNQRPSGVPDRFSGSKSGTSASLAISGLRSEDEADYYCAAWDDSLSV
AAO49730         TQPPS-ASGTPGQRVTISCSGSSSSIGSNTVNWYQHLPGTAPKLLIYSNNERPSGVPDRFSGSKSGTSASLAISGLQSEDEADYYCASWDDSLNG
AAP31886     QSVLTQPPS-ASGTPGQRVTISCSGSTSNIGTNYVYWYQQLPGTAPKLLIYRNNQRPSGVPDRFSASKSGTSASLAISGLRSDDESDYYCAAWDDSLRV
AAW67417     SYELTQPPS-ASGTPGQRVTISCSGSSSNIGSNYVYWYQQLPGTAPKLLIYRNNQRPSGVPDRFSGSKSGTSASLAISGLRSEDEADYYCAAWDDSLSG

Z22191       QSVLTQPPS-VSAAPGQKVTISCSGSSSN-IGNNYVSWYQQLPGTAPKLLIYDNNKRPSGIPDRFSGSKSGTSATLGITGLQTGDEADYYCGTWDSSLSA
CAA70558     QSALTQPPS-MSAAPGQKVTFSCSGRSSN-IGNNYVSWYQQLPGTAPKLLIYDNNKRPSGIPDRFSGSKSGTSATLGITGLQTGDEADYYCGTWDSSLGA
AAY33437     QSVVTQPPS-VSAAPGQKVTISCSGSSSN-IGNNYVSWYQQLPGTAPKLLIYENNKRPSGIPDRFSGSKSGTSATLGITGLQTGDEADYYCGTWDGSLSG
AAY33458     QSVVTQPPS-VSAAPGQKVTISCSGSSSN-IGDNYVSWYQQLPGTAPKLLIYDNNKRPSGIPDRFSGSKSGTSATLDITGLQAGDEADYYCGTWDSSLSA
AAX57569     ----TQPPS-VSAAPGQKVTISCSGSSSN-IGNNYVSWYQQLPGTAPKLLIYDNNKRPSGIPDRFSGSKSGTSATLGITGLQTGDEADYYCGTWESSLSA
CAD30279     QSVLTQPAS-VSGSPGQSITISCSGSSSN-IGNNYVSWYRQLPGTAPKLLIYDNNKRPSGIPDRFSGSKSGTSATLGITGLQTGDEADYYCGTWDSSLSA
CAA54621     QSVLTQPPS-VSAAPGQKVTISCSGNSSN-IGNNYVSWYQHLPGTAPKLLIYDNNKRPSSIPDRFSGSKSGTSATLGITGLQTGDEADYYCATWTSILRV
AAR88382     QSVLTQPPS-VSAAPGQKVTISCSGSSSN-IGNNDVSWYQQFPGTVPKLVIYENNQRPSGIPDRFSGSKSGTSATLGITGLQTGDEADYYCGTWDSSLSA
AAC06030     QTVVTQEPS-LTVSPGGTVTLSCSGSSSN-IGNNYVSWYQQLPGTAPKLLIYENNKRPSGIPDRFSGSKSGTSATLGITGLQTGDEADYYCGTWDSSLSA

Z22197       QSALTQPAS-VSGSPGQSITISCTGTSSDVGGYNYVSWYQQHPGKAPKLMIYEVSNRPSGVSNRFSGSKSGNTASLTISGLQAEDEADYYCSSYTSSSTL
```

```
AAB68782      QSALTQPAS-VSGSPGQSITISCTGTSSDVDGYNYVSWYQQYPGKAPKLMIYEVSNRPSGVSNRFSGSKSGNTASLTISGLQAQDEADYYCCSYTSSSTL
AAY33419      QSALTQPAS-VSGSPGQSITISCTGTSSDVGSYNLVSWYQQHPGKAPKLMIYEGSKRPSGVSNRFSGSKSGNTASLTISGLQAEDEADYYCSSYTSSSTL
CAD30277      HVILTQPAS-VSGSPGQSITISCTGTNSDVGGYNYVSWYQQHPGEAPKLMIYEVTNRPSGVSNRFSGSKSGNTASLTISGLQAEDEADYYCSSYASSTTL
AAY33385      QSALTQPAS-VSGSPGQSITISCTGSRSDVGSYNLVSWYQQHPGKAPKLMIYEGSKRPSGVSNRFSGSKSGNTASLTISGLQAEDEADYYCSSYTSSSTL
AAS19431      QSALTQPAS-VSGSPGQSITISCTGTSSDLGGHNFVSWYQQHPGKAPKLMIYDVFNRPSGVSSRFSGSKSGNTASLTISGLQAEDEADYFCSSYTITNIV
CAI99668.1    -----QPAS-VSGSPGQPITISCTGTSSDVGDYNYVSWYQQHPGKAPKLMIYDVSNRPSGVSNRFSGSKSANTASLTISGLQAEDEADYYCCSYTTVS
ACF34450.1    -----QPAS-VSGSPGQSVTISCTGTSSDVGLYNYVSWYQQHPGKAPKLLIYDVTNRPSGVSNRFSGSKSGNTASLTISGLQAEDEADYYCGSYTSNI
ABP48425.1    -----QPAS-VSGSPGQSITISCTGTSSDVGSYNLVSWYQQHPGKAPKLMIYEVSKRPSGISNRFSGSKSGNTASLTISGLQAEDESDYYCCSYAGSS
AAB37390.1    -----QPAS-VSGSPGQSITISCTGTRRDVGGYNFVSWYQQHPGKAPKLMIYEVSHRPSGVSTRFSASKSGSTASLTISGLQAEDEADYYCSSYSSTT
ABI73997.1    -----QPPS-VSGSPGQSVTISCTGTSSDVGGYNYVSWYQLHPGKAPKLMIYDVSKRPSGVPDRFSGSKSGNTASLTISGLQAEDEADYYCCSYAGSY
AAF20498.1    -----QPAS-VSGSPGQSITISCTGTSSDVGNYNLVSWYQQHPGEAPKLMIYEDSKRPSGVSNRFPGSKSGNTASLTVSGLQAEDEADYYCCSYAGSS
AAD03781.1    -----QPAS-VSGSPGQSITISCTGTSSDVGGSGYVSWYQHHPGKAPKLMIYEVNHRPSGVSNRFSGSKSGNTASLTISGLQPEDEADYHCISYTSRS
CAB38070.1    -----QPAS-VSGSPGQSITISCTGTSSDVGSYNLVSWYQHHPGKAPKLMIYDVNKRPSGVSDRFSGSKSGNTASLTISGLQAEDEGDYYCCSYAGSS
AAK94857.1    -----QPRS-VSGSPGQSVTISCTGTSSDVGGYNYVSWYQQHPGKAPKLMIYDVSKRPSGDPDRFSGSKSGNTASLTISGLQAEDEADYYCCSYAGSY
AAD44120.1    -----QPDS-VSGSPGQSITISCTGTSSDVAIYNYVSWYQQYPTKAPKLTIYEVNNRPSGVSNRFSGSKSGNTASLTISGLQAEDEADYYCSSYTITG

Z22208        SYELTQPPS-VSVSPGQTASITCSGDKLGDKYACWYQQKPGQSPVLVIYQDSKRPSGIPERFSGSNSGNTATLTISGTQAMDEADYYCQAWDSSTA
AAY33341      SYVLTQPPS-VSVAPGQTASITCSGDKLGDKYASWYQQKPGQSPVLVIYQDSKRPSGIPERFSGSNSGNTATLTISGTQAMDEADYYCQAWDSSSL
AAC43025      SYELTQPPS-VSVSPGQTASITCSGDKLGDKYVCWYQQKPGQSPLLVIYQDTKRPSGIPERFSGSSSENTATLTISGTQAMDEADYYCQAWDTNTA
AAB48972      --ELTQSPS-VSVSPGQTVSITCSGDKLGDKYACWYQQKPGQSPVLLIYQDSKRPSGIPERFSGSNSGNTATLTISGTQAMDEADYYCQAWNSSTV
PC4283        --ELLQPPS-VSVSPGQTASITCSGDKLGDKYTCWYQQKPGQSPVLVIYQDNKRPSGIPGRFSGSNSGNTATLTISGTQAMDEADYYCQAWDSNRN
AAO49735      ----DSPPS-VSVSPGQTASITCSGDKLGDKYASWYQQKPGQSPVLVIYQDSKRPSGIPERFSGSNSGNTATLTISGTQAMDEADYYCQAWDSSTW
AAY33424      SYVLTQPPS-VSVSPGQTASITCSGDKLGDKYASWYQQRTGQSPVVVIYQDTKRPSGIPERFSGSNSGNTATLTISGTRPMDEADYYCQAWDSSTV
AAD16651      -----QPPS-VSVSPGQTASFTCSGDKLGNKYASWYQQRPGQSPVLVIYQDAKRPSGIPDRFSGSTSGNTATLTISGTQAMDEADYYCQAWDNTHV
CAB54548      SFELIQPPS-VSVSPGQTAIITCSGDKLGDKYASWYQQKPGQSPILVISRDNRRPSGIPERFSGSNSGNTATLTISGAQTIDEAEYYCQAWDSTT
AAC16878      SYDLTQPPA-VSVSPGQTASISCSGHKLEDKYACWYQQKPNQSPVLIVYEDKKRPTGIPERFSGSNSGNTATLNISGAQALDEADYYCQAWDRTTV
ABA71475      SSELTQPAS-VSVSPGQTASITCSGDKLRDKYASWYQQKPGQSPVLVIFQDAKRPSGIPERFSGSTSGDTATLTISGTQATDEADYYCQAWDSSSV
ABA71587      SYELTQPPS-VSVSPGQTASITCSGETLGDKYASWYQQKPGQSPVLVIHEDNRRPSGIPERFSASNSGNTATLTISGTQSMDEADYYCQAWGRTTV
AAC16865      SYDLTQPPS-VSVSPGQTVTITCSGYNLGNKYACWYQQKLGQSPVLVIYQDTKRPSGIPERFSGANSGNTATLTITGTQTMDEADYYCQTWDSSTP
AAC16823      SYELTQPPS-VSVSPGQTANIICSADKLGDKYASWYQKKPGQSPLLVISQDTKRPSGIPERFSGSNSGNTATLTISGTQAMDEADYFCQAWDRSTV
AAK94863      SYELTQPPS-VSVSPGQTASITCSGDKLGNKYTSWYRQKPGQPPVLVIFQDTKRPSGIPERYSGSNSGNTASLTISGTQAMDEADYYCQAWDSNSW
CAA40953      SYELTQPPS-VSVSPGKTASITCSGDKLGDKYASWYQQKAGQSPVLVIYRHSKRPSGIPERFSGSNSGNTATLTISGTQVMDEADYYCQAWDSSIV
AAB34387.1    SYELTQPPS-VSVSPGKTASITCSGDKLGDKYACWYQQKPGQSPVLVIYQDSKRPSGIPERFSGSNSGNTATLTISGTQAMDEADYYCQAWDSST
Pir           --ELLQPPS-VSVSPGQTASITCSGDKLGDKYTCWYQQKPGQSPVLVIYQDNKRPSGIPGRFSGSNSGNTATLTISGTQAMDEADYYCQAWDSNR
AAC43025.1    SYELTQPPS-VSVSPGQTASITCSGDKLGDKYVCWYQQKPGQSPLLVIYQDTKRPSGIPERFSGSSSENTATLTISGTQAMDEADYYCQAWDTNT
AAC15214.1    SYELTQPPS-VSVSPGQTASITCSGEKWGDKYASWYQQKPGQSPVLVIYQDSKRPLGIPERFSGSKSGKTATLTISGTQAMDEADYYCQAWDSRK

Z73673        NFMLTQPHS-VSESPGKTVTISCTRSSGSIASNYVQWYQQRPGSSPTTVIYEDNQRPSGVPDRFSGSIDSSSNSASLTISGLKTEDEADYYCQSYDSSN
AAD23181.1    --------S-VSESPGKTVTISCTRSSGSIASIYVQWYQQRPGSSPTTVIYEDNQRPSGVPDRFFGSVDSPSNSASLTISGLKTEDEADYYCHTYNNTN
AAD23175.1    --------S-VSESPGKTVTISCTGSSGSIADNYVQWYQQRPGSAPTSVIYEDNHRPSGVPDRFSGSIDSPSNFASLTIFGLKTEDEADYYCQSFDNHN
AAD23183.1    --------S-VSGSPGKTVTISCSVNSGNIASNYVQWYQQRPDNAPNIVIFEDDQRPSGVPDRFSGSIDISSNFASLTIFGLSPEDEADYYCQSYHDTT
AAD23184.1    --------S-VSGSPRKTVTISCSVNTGNIASNYVQWYQQRPDNAPNIVIFEDDQRPSGVPDRFSGSIDISSNFASLTIFGLSPEDEADYYCQSYHDTT
AAR03000.1    --------S-VSESPGKTVTISCTRSSGSIASDYVQWYQQRPGSAPTTVIYEDNQKPSGVPDRFSGSIDSSSNSASLTISGLKAEDEADYYCQSYDSSN
AAR02935.1    ----------VSESPGKTVTISCTRSSGSIASNYVQWYQQRPGSAPTAVIYKDNQRPSGVPDRFSGSIDSSSNSASLTISGLKTEDEADYSCQSYDTSS
```

```
CAE18302.1   -------HS-VSGSPGQTVTISCARSSGSIANSFVQWYQLRPGSTPTTVIFEDDQRPSGVPDRFSGSVDGSSNVASLTISGLKIEDEADYFCQSYDATI
ABA1670.1    -------------SDFGRGNISCTRSSGGITSNYVQWYQQRPGSSPTTVIYEDDQRPSGVPDRFSGSIDTSSNSASLTISGLKTEDEADYHCQSYDISN
BAC01822.1   -------HS-VSESPGKTVTISCTRSSGSIASNYVQWYQQRPGSAPTTVIYEDNQRPSGVPDRFSGSIDSSSNSASLTISGLKTEDEADYYCQSYDSSK
BAC01823.1   --------S-VSESPGKTVTISCTRSSGSIASNYVQWYQQRPGSSPTTVIYEDNQRPSGVPDRFSGSIDSSSNSASLTISGLKTEDEADYYCQSYDSSN
BAC01869.1   --------S-VSESPGKTVTISCTRSSGSIASNYVQWYQQRPGSAPTTVIYEDNQRPSGVPDRFSGSIDSSSNSASLTISGLKTEDEADYYCQSYDSSN
ABU90660.1   -----QPHS-VSESPGKTVTISCTGSSGSFVSNYVQWYQQRPGSAPITVIYEDNQRPSGVPDRFSGSIDRSSNSASLTISGLKTEDEADYYCQSYDSSN
AAF21612.1   -----QPHS-VSESLGKTVTISCTRAGGSIASNYVQWYQQRPGSSPTTVIYEDNQRPFGVPDRFSGSIDTSSNSASLTISGLKTEDEADYYCQSYDSEN
BAC01857.1   --------S-VSESPGKTVTISCTRSSGSIASNYVQWYQQRPGSAPTTVIYEDNQRPSGVPDRFSGSIDSSSNSASLTISGLKTEDEADYYCQSYDSSK
BAC01863.1   -----------SESPGKTVTISCTRSSGSIASNYVQWYQQRPGSSPTTVIYEDNQRPSGVPDRFSGSIDSSSNSASLTISGLKTEDEADYYCQSYDSSN
AAD44136.1   -----QPHS-VSESPGKTVTISCTRSSGSIASTYVQWYQQRPGRAPTTVIFEDNERPSGVPDRFSGSIDTSSNSASLTISGLESEDEADYFCQSYDDNS
CAC29404.1   ------QPHSVSESPGKTVTISCTGSSGSIADNYVQWYQQRPGSAPTTVLYEANQRPSGVPGRFSGSIDTSSNSASLTISGLETEDEADYYCQSFDGSN
ABC66897.1   -------------SPGKTVTISCTRSSGSIASNYVQWYQQRPGSSPTTVIYEDNQRPSGVPDRFSGSIDSSSNSASLTISGLKTEDEADYYCQSYDSSN
CAA85265.1   -------------SPGKTVTISCTRSSGSIASNYVQWYQQRPGSAPTTVIYEDNQRPSGVPDRFSGSIDSSSNSASLTISGLKTEDEADYYCQSYDSSN
AAM46260.1   -------------SPGKTVTISCTRSSGSIASNYVQWYQQRPGSSPTTVIYEDNQRPSGVPDRFSGSIDSSSNSASLTISGLKTEDEADYYCQSYDSST
AAY33432.1   NFMLTQPHS-VSESPGKTVTISCTRSSGSIASNYVQWYQQRPGSAPTTVIYEDNQRPSGVPDRFSGSIDSSSNSASLTISGLKTEDEADYYCQSYDSS-
ABU90623.1   -----QPHS-VSESPGKTVTISCTRSSGNIASNYVQWYQQRSAPITVIYEDNQRPSGVPDRFAGSIDRSSNSASLTISGLKTEDEADYYCQSYDARNVV
ABU90610.1   -----QPHA-VSESPGKTVIISCTRSSGSIGSNYVQWYQQRPGRSPSTVIYEDIERPSGVSDRFSGSIDQSSNSASLTISGLKAEDEADYYCQSYDSHD
CAJ75554.1   ------------ESPGKTVTISCTRSSGSIASNYVQWYQQRPGSAPSTVIYEDDQRPSGVPDRFSGSIDSPSNSASLTISGLRTEDEADYYCQSYDSTN
AAY33406.1   -----QPHS-VSESPGKTVTISCTRSSGSIASNYVQWYQQRPGSAPTTVIYEDNQRPSGVPDRFSGSIDSSSDSASLTISGLKTEDEADYYCQSYDSSN
AAD16784.1   -----------SESPGKTVSISCTRSSGYIASNFVQWYQQRPGRAPTTVIYEDKQRPSGVPDRFSGSIDTSSNSASLTISGLKTEDEADYYCQSYDSSN
AAD23180.1   --------S-VSESPGKTVTISCTRSSGSIASIYVQWYQQRPGSSPTTVIYEDNQKPSGVPDRFFGSVDGSSNSASLTISGLKTEDEADYYCHTYNNTN
AAM92096.1   -----QPHS-VSESPGKTVTISCTGSSGSIASHYVQWYQQRPGSAPTTIIYEDNQRPSGVPDRFSGSIDSSSNTASLTISGLKTEDEADYYCQSYDNSN
AAV64253.1   --------S-VSESPGKTVTISCTGSSGSIASNYVQWYRQRPGSAPTTVIYEDNQRPSGVPDRFSGSIDSSTNSASLTISGLKTEDEADYYCQSYDSSN
AAD23171.1   --------S-VSESPGKTVTISCTGSSGSIASKYVQWYQQRPGSTPTTVIYEDNQRPSGVPDRFSGSIDSSSNFASLTIFGLKTEDEADYYCQSYDSTN
AAX57538.1   -----QSPS-VSESPGKTITISCTRSSGSIASSYVQWYQQRPGSAPTTVIYEDSQRPSGVPDRFSGSIDISSKSASLTISGLKTEDEADYYCQSYDSHS
AAD23175.1   --------S-VSESPGKTVTISCTGSSGSIADNYVQWYQQRPGSAPTSVIYEDNHRPSGVPDRFSGSIDSPSNFASLTIFGLKTEDEADYYCQSFDNHN
```
